# Angiogenesis-related gene signatures reveal the prognosis of cervical cancer based on single cell sequencing and co-expression network analysis

**DOI:** 10.3389/fcell.2022.1086835

**Published:** 2023-01-12

**Authors:** Jiawen Kang, Xiaoqing Xiang, Xiaoyan Chen, Jingwen Jiang, Yong Zhang, Lesai Li, Jie Tang

**Affiliations:** ^1^ Department of Internal Medicine, Medical College of Hunan Normal University, Changsha, Hunan, China; ^2^ Department of Pathology, Hunan Cancer Hospital/the Affiliated Cancer Hospital of Xiangya School of Medicine, Central South University, Changsha, Hunan, China; ^3^ Department of Gynecologic Oncology, Hunan Cancer Hospital/the Affiliated Cancer Hospital of Xiangya School of Medicine, Central South University, Changsha, Hunan, China

**Keywords:** cervical cancer, single cell sequencing, prognostic models, angiogenesis-related genes, immunotherapy

## Abstract

Cervical cancer ranks first in female reproductive tract tumors in terms of morbidity and mortality. Yet the curative effect of patients with persistent, recurrent or metastatic cervical cancer remains unsatisfactory. Although antitumor angiogenic drugs have been recommended as the first-line treatment options for cervical cancer, there are no comprehensive prognostic indicators for cervical cancer based on angiogenic signature genes. In this study, we aimed to develop a model to assess the prognosis of cervical cancer based on angiogenesis-related (AG) signature genes, and to provide some reference for the comprehensive treatment of cervical cancer in the clinical setting. First we screened the AG gene set from GeneCard website, and then performed angiogenesis-related scores (AGS) per cell from single cell sequencing dataset GSE168652, followed by performing weighted gene co-expression network analysis (WGCNA) for cervical cancer patients according to angiogenesis phenotype. Thus, we established a prognostic model based on AGS by taking the intersection of WGCNA angiogenic module gene and differential gene (DEGs) of GSE168652. The GSE44001 was selected as an external validation set, followed by performing ROC curve analysis to assess its accuracy. The results showed that we successfully constructed a prognostic model related to the AG genes. Patients in the high-AGS group in both the train, test and the validation sets had a worse prognosis than those in the low-AGS group, had lower expression of most immune checkpoint-associated genes and lower tumor mutational burden as well. Patients in the low-AGS group were more sensitive to AMG.706, Bosutinib, and Lenalidomide while Imatinib, Pazopanib, and Sorafenib were more recommended to patients in the high-AGS group. Finally, *TXNDC12* and *ZC3H13*, which have high hazard ratio and poor prognosis in the model, were highly expressed in cervical cancer cell lines and tissue. Meanwhile, the results showed that *TXNDC12* promoted the migration of cervical cancer cells and the tubule-forming ability of endothelial cells. In conclusion, our model based on genes with AG features can effectively assess the prognosis of cervical cancer, and can also provide reference for clinicians to choose immune-related treatments.

## Introduction

According to the Global Cancer Data 2021, cervical cancer has the 4th incidence and mortality rate infemale tumors worldwide (Sung et al., 2021) and the 1st incidence in female reproductive tract tumors, with a high prevalence in developing countries and poor regions. The two main histological subtypes of cervical cancer include cervical squamous cell carcinoma, which accounts for approximately 80% of all cases of cervical cancer, and cervical adenocarcinoma. The use of HPV vaccine has reduced the risk of cervical cancer (Cohen et al., 2019). With the widespread availability of screening and improved screening strategies, patients with early-stage cervical cancer are being diagnosed in a timely manner. Patients with early-stage cervical cancer are mainly treated with radical hysterectomy, with locally advanced stages are treated with concurrent chemoradiotherapy, can achieve better prognosis. However, for patients with persistent, recurrent and metastatic cervical cancer, the current treatment options are limited and the prognosis is poor. It is urgent to explore the mechanisms that lead to metastasis or recurrence of cervical cancer.

Angiogenesis is a regulatory process in which cytokines, receptors and molecules are involved to function through different signaling pathways in physiological or pathological conditions (Sajib et al., 2018). Angiogenesis is triggered by pro-angiogenic molecules released from tumor cells and promotes tumor growth and metastasis to drive tumor progression in turn (Viallard and Larrivée, 2017). For cervical cancer, bevacizumab (a representative antiangiogenic agent) has been admitted as first-line regimens for recurrent or metastatic cervical cancer which has been endorsed by National Comprehensive Cancer Network (NCCN) guidelines. However, current treatment regimens for patients with cervical cancer can’t offer modest improvements in progression-free survival (PFS) and overall survival (OS) (Ramjiawan et al., 2017; Watson and Al-Samkari, 2021). It has been suggested that the single use of targeting anti-angiogenic agents can activate or substitute growth factor signaling pathways thereby creating redundancy, which can instead lead to the development of drug resistance (Ribatti, 2011). Therefore, in order to avoid resistance to anti-angiogenic therapy, multiple anti-angiogenic molecular agents or their combination with other therapeutic regimens has been emphasized (Ribatti et al., 2021). Here, exploring models to assess the prognosis of cervical cancer based on the set of AG signature genes would provide new suggestion for anti-vascular targeted therapy. With the continuous advancement of scientific and technological tools, the application of sequencing methods has brought a qualitative leap forward in the field of oncology research. Transcriptome analysis is an important part of current genetic research, among which bulk RNA-seq is widely used for transcriptome RNA sequencing (Hong et al., 2020). Although bulk RNA-seq can help us to explore differential expression of genes related to vascular neoplasia, it can only reveal the difference in gene expression at the histological level. Whereas single-cell sequencing has the unique advantage of revealing all intracellular gene expression differences (Hwang et al., 2018). Here, we combined single-cell sequencing data and bulk RNA-seq data for analysis.

As we know, the tumor microenvironment consists of tumor cells, stromal cells, and immune cells. In the tumor microenvironment, immune cells are affected by angiogenesis and secrete a large number of molecules to promote angiogenesis (Ribatti and Crivellato, 2009). Neovascularization serves as a bridge among different cells and plays vital role between intercellular communications. Such as vascular endothelial growth factor A (*VEGFA*), the main pro-angiogenic factor, could cause immunosuppression (Albini et al., 2018). The efficacy of anti-angiogenic drugs in combination with immune checkpoint inhibitors is also attributed to the crosstalk of immune cells in the tumor microenvironment (Ribatti et al., 2021). It has been shown that angiogenesis-related gene signatures in gastric cancer (Ren et al., 2020; Qing et al., 2022), hepatocellular carcinoma (Lv et al., 2021), and squamous carcinoma of multiple tissue types (Qin et al., 2021)can predict patient prognosis and elucidate the characteristics of the immune microenvironment to guide immunotherapy. Therefore, studying immune infiltration based on AG genes in cervical cancer can help to provide new predictive prognostic markers and reference for comprehensive treatment.

## Materials and methods

### Data acquisition

Transcriptional profiles and clinical information of 309 cervical cancer patients were obtained from the cervical squamous cell carcinoma and endocervical adenocarcinoma (CESC) cohort of the Cancer Genome Atlas (TCGA, https://portal.gdc.cancer.gov/), excluding the “-11A” normal samples and reserving the “-01A” tumor samples. After screening and matching the clinical data, 283 samples were selected as the TCGA-total set. The data set GSE44001 (Lee et al., 2013) of cervical cancer patients (*n* = 300) was selected from the Gene Expression Omnibus database (GEO, https://www.ncbi.nlm.nih.gov/geo/) as the validation set. All data were log2 transformed. The single-cell sequencing dataset GSE168652 (Li et al., 2021) was selected from GEO. A total of 5,098 genes related with angiogenesis (AG genes) were downloaded from the GeneCard website (https://www.genecards.org/) by the keyword “angiogenesis”, and 1,245 angiogenesis genes (AG-gene set) were included by sorting the correlation score >1. Immunohistochemical staining (IHC) data of ZC3H13 gene was obtained by the Human Protein Altas (HPA) (https://www.proteinatlas.org/).

### Single-cell sequencing data processing

The “Seurat” package (Butler et al., 2018) was used for quality control of the single-cell sequencing data. Cells with less than 10% of mitochondrial genes, less than 5% of hemoglobin genes, less than 30% of ribosomal genes, and a gene number expression range of 200–3,000 were retained. The number of highly variable genes was set to 3,000 and integrated by SCT correction. Then, the “DIMS” parameter was set to 20, and the data were downscaled using the tSNE method and clustered by the “KNN” method. In the single-cell sequencing data, the scores of AG gene-sets were calculated by applying the “PercentageFeatureSet” function, and the single-cell sequencing data was divided into high and low-AGS cell groups according to the median value.

### Construction and evaluation of prognostic models

The ssGSEA algorithm was used to calculate the scores associated with the AG gene-set for each patient from TCGA-CESC. Each patient was screened using variance, and the top 90% of valid genes were selected, and the “WGCNA” package (Langfelder and Horvath, 2008) was called for analysis to select the gene modules (*p*-value < 0.05) for the phenotypic association with scores of AG gene-set. Next, prognosis-related AG genes were obtained by univariate COX analysis. Subsequently, using the R package “glmnet” (Friedman et al., 2010)was performed to further filter out eight genes, and the regression coefficients of each gene were calculated to construct a prognostic model. Based on the prognostic model, the AGS of each patient sample was calculated, and then patients were divided into two groups: high AGS group and low AGS according to the median value (AGS-patient). The area under the ROC curve was observed by “timeROC” package (Blanche et al., 2013) and “survivalROC” package (Heagerty et al., 2000).

### Immune infiltration analysis and differential protein analysis

For the immune infiltration analysis, “IOBR” package (Zeng et al., 2021) was download. We compared CIBERSORT(Chen et al., 2018), EPIC (Racle and Gfeller, 2020), MCP_counter (Becht et al., 2016), xCell (Aran et al., 2017), TIMER (Li et al., 2017), and Quanti-seq (Plattner et al., 2020)algorithms. Also, the differential expression of immune checkpoint genes and major markers for immunotherapy in patients was observed between the high- and low-AGS groups. The mutations data in cervical cancer were downloaded from Cbioportal (https://www.cbioportal.org/datasets) and visualized by the “maftools” package (Mayakonda et al., 2018). The top 20 tumor mutations with the highest mutation frequency between high- and low AGS-groups were visualized. RPPA protein microarray data (van Dijk et al., 2022) for cervical cancer were downloaded from the TCPA website (https://www.tcpaportal.org/).

### Construction of nomogram

The “rms” package was used to construction a nomogram, and ROC curves were performed to evaluate the predictive accuracy of nomogram. DCA decision analysis was used to evaluate the predictive accuracy of the prognostic model.

### Cell culture and RT-qPCR

Human normal cervical cell lines (ECT1/E6E7) and human cervical cancer cell lines (SiHa, Hela cells) were cultured in DMEM medium (Procell, Wuhan, China) containing 1% Penicillin and Streptomycin (BI, Israel) and 10% fetal bovine serum, and human cervical cancer (CaSki cells) and Human umbilical vein endothelial cells (HUVECs) were cultured in RPMI-1640 medium under the same conditions at 37°C and 5% CO_2_. The cells were trypsinized and seeded into 6-well plates, washed over with PBS after 80%–90% growth, added TRIzol reagent (Vazyme, Nanjing, China) and blown down and collected. Add chloroform to each tube and mix upside down, let stand and centrifuge at 4°C and 12,000 rpm for 15 min, carefully take the upper aqueous phase and transfer to a new centrifuge tube for subsequent experiments. After adding the same volume of isopropanol and mixing upside down, centrifuge again, aspirate the supernatant and add 75% alcohol prepared with DEPC water, centrifuge and pour off the supernatant, dry the RNA. 1ul of RNA was detected by spectrophotometer, and the A260/280 values were above 1.8. The qPCR reaction system was prepared by using reverse transcription and quantification kit (PerfectStart Uni RT&Qpcr Kit, AUQ-01, China) for reverse transcription. The qPCR reaction system was placed on a fluorescence qPCR instrument, and the △CT, △△CT value, 2^-△△CT^ value of each sample was calculated. The primers were:

ZC3H13-F:CAGAGGTGACAGAAGCAGAGCATAC,

ZC3H13-R: GCA​GCA​GTA​GTG​GCA​GCA​AGA​G;

TXNDC12-F: TCC​TGC​TCC​TCG​TCA​TCT​CTT​CTG,

TXNDC12-R: AGC​TGC​TTC​TTT​CTT​CCC​ATC​TTC​C;

GAPDH-F: CAG​GAG​GCA​TTG​CTG​ATG​AT,

GAPDH-R:GAAGGCTGGGGCTCATTT.

#### Immunohistochemistry

We collected paraffin sections of patients with non-cancer or cancer from Hunan Cancer Hospital and performed immunohistochemical staining. Then Samples were dewaxed with ethanol and blocked to inhibit endogenous peroxidase activity. They were retrieved antigen by autoclave boiling for 20min. Samples were incubated overnight at 4°C with rabbit anti-CD31 (ZEN-BIOSCIENCE, Chengdu, China, 1:100), anti-S100A9 (Abclonal, Wuhan, China, 1:100), anti-TXNDC12 (Abclonal, Wuhan, China, 1:100), followed by incubation with the secondary antibody PV-9000 Kit (zsbio, Beijing, China) at 37°C for 20 min. Cell nuclei were stained blue with use of hematoxylin. The ImageJ Software 1.53 (United States) was used to analyze protein expressions and perform statistics.

#### Western blotting

Proteins were first extracted by adding RIPA lysate and protease inhibitor PMSF(Servicebio, Wuhan, China), denatured with SDS for 10 min using 15% of the separation gel and concentrated gel were run in electrophoresis solution at a constant voltage of 80V–120 V for 80 min. Membranes were rotated at a constant current of 260 mA for 90 min. Then incubation were performed in skimmed milk at 37°C for 2 h. Membranes were incubated overnight at 4°C with rabbit anti-TXNDC12 (Abclonal, Wuhan, China, 1:1,000), followed by incubation with the secondary antibody (Bioworld, United States, 1:10000) at 37°C for 60 min.

#### Tubule formation experiment

Cervical cell lines SiHa and CaSki were transfected with sh-*TXNDC12*, the negative control (sh-NC) for 48 h (Genechem, Shanghai, China). And after 48 h the supernatant of cell was taken to act on endothelial cells for tubule formation assay. 96-well plate was coated with 50 μl Matrigel each well for 1 hour to solidify. Thereafter, HUVECs were seeded into wells (8,00000 cells/well), and 100 μl supernatant (CM) from transfection-pretreated tumor cells. Photographs were taken after 4h and 8 h to observe the tube formation ability. Tube formation was quantified using ImageJ (ImageJ software, United Ststes).

#### Scratch wound healing assay

Wound healing assays were used to assess the migration of SiHa and CaSki cell lines. Cells were transfected with sh-*TXNDC12* plasmids using the ExFect Transfection Reagent (Vazyme, China) for 48 h for subsequent experiments. When the cell density in the 12-well plate grows to 80%–90%, we use the sterile tip of a pipette to draw a vertical line straight down the center of the 12 wells, taking care to keep the same width in each well. The area of the cell scratch was taken pictures to record when the line is drawn. In 24 h later, the cell scratch area was taken pictures again to record. Cell migration rate/Wound Healing = (0 h scratch width—24 h scratch width)/0 h scratch width * 100%. Scratch Wound Healing Assay was quantified using ImageJ (ImageJ software, United States).

#### GESA pathway and functional enrichment analysis

In gene set enrichment analysis (GSEA), The GSEA software (version 3.0) was obtained from GSEA (DOI:10.1073/pnas.0506580102, http://software.broadinstitute.org/gsea/index.jsp) website. The samples were divided to high-group (≥50%) and low-group (<50%) based on expressed level of TXNDC12, and downloaded c2. cp.kegg.v7.4. symbols.gmt subset from Molecular Signatures Database (DOI:10.1093/bioinformatics/btr260, http://www.gsea-msigdb.org/gsea/downloads.jsp). We set a minimum gene set of 5, a maximum gene set of 5,000, and one thousand re-samplings, *p*-value < 0.05 and FDR < 0.25 was considered statistically significant. Kyoto Encyclopedia of Genes and Genomes (KEGG) set API (https://www.kegg.jp/kegg/rest/keggapi.html) was used to obtain the gene annotations of the KEGG pathway. R package “clusterProfiler” was used for enrichment analysis.

#### Screening small molecule drugs and drug sensitivity analysis

In the Drug Signatures Database (DSigDB, http://tanlab.ucdenver.edu/DSigDB), we included and screened eight model genes for corresponding small molecule drugs (Supplementary Table S1). Based on the score, six potential small molecule drugs were listed (*p*-value < 0.05). Drugs with high scores could be related with treatments of cervical cancer patients by regulating angiogenesis. The Genomics of Drug Sensitivity in Cancer (GDSC, http://www.cancerrxgene.org/) Database was used to compare the difference in the half-maximal inhibitory concentration (IC50) of drugs between high-AGS group and low-AGS group. R package “pRRophetic” was used for drug sensitivity analysis. *p*-value < 0.05 was considered statistically significant.

#### Statistical analysis

Data analysis was performed in R version 4.2 (https://www.r-project.org). The experimental data was statistically analyzed using GraphPad Prism 8 software. T-test was used to compare the data between the two groups. *p*-value < 0.05 was indicated a statistically significant difference.

## Result

### A flowchart of the study

**FIGURE 1:**
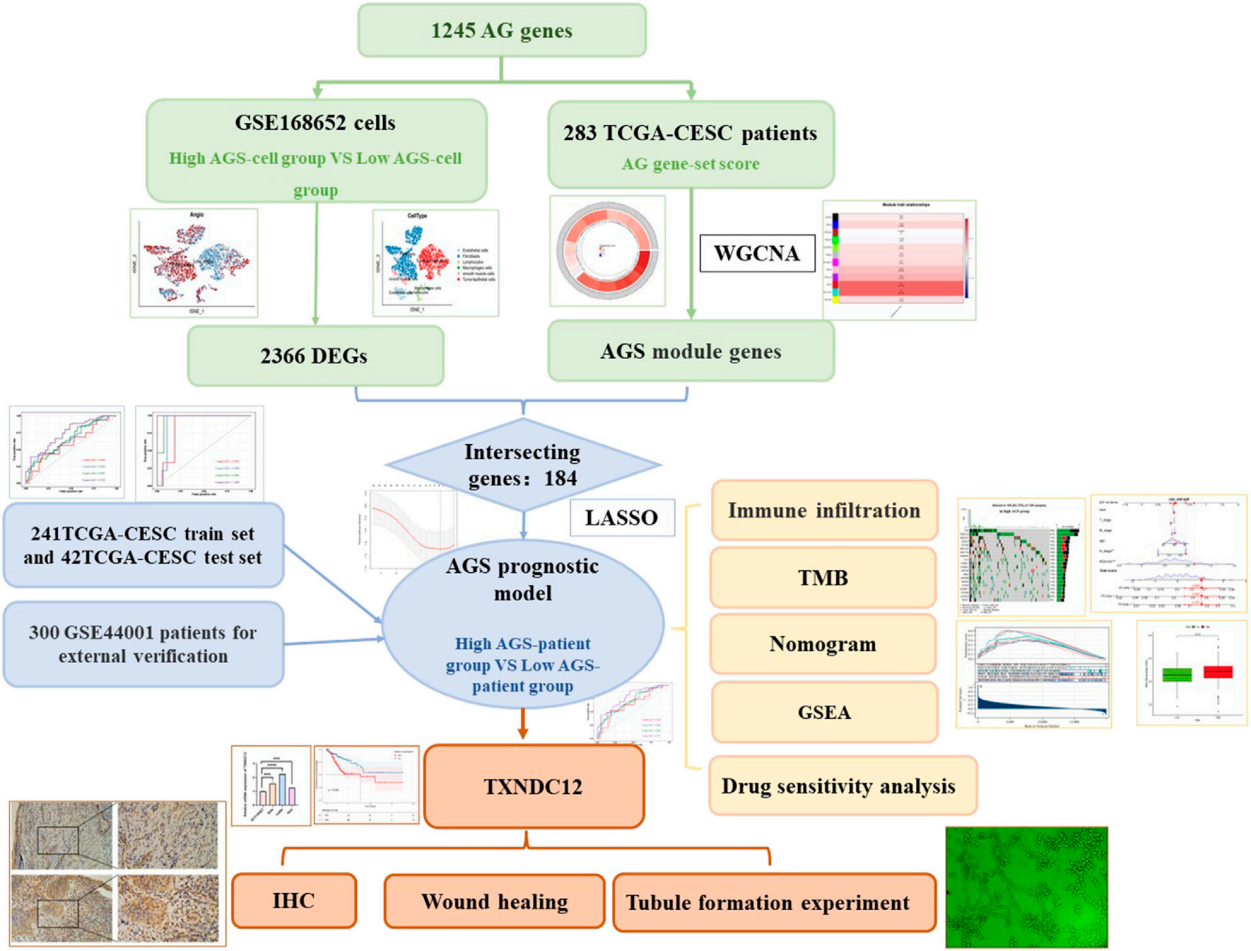
Flowchart of the study.

The flowchart of establishing a prognostic model based on angiogenesis-related gene signatures was shown in Figure 1.

#### Identification of different genes in single-cell sequencing dataset and weighted co-expression network analysis for patients in cervical cancer

**FIGURE 2:**
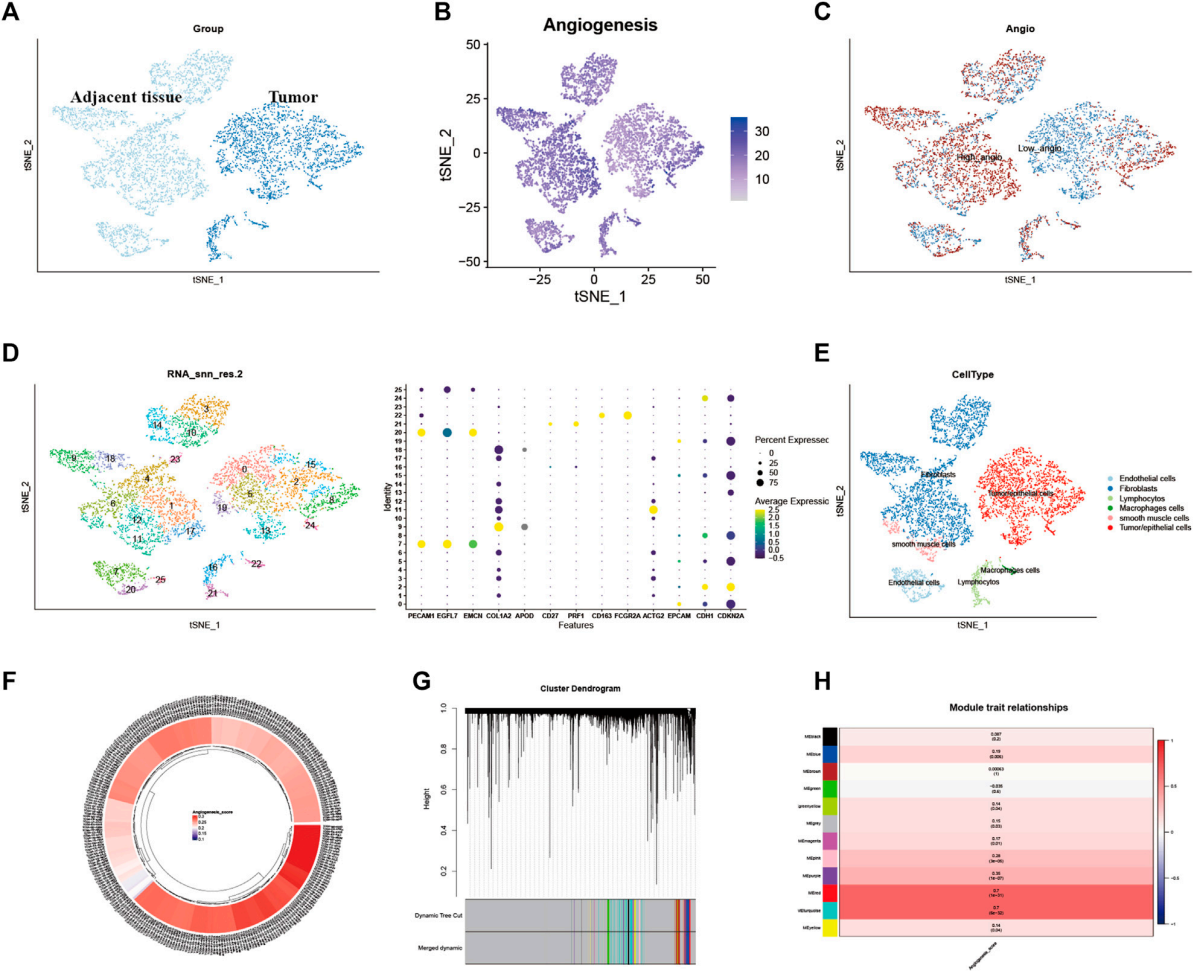
Identification of Angiogenesis-Related Different Genes and Selection of Module Genes by WGCNA. **(A)**. Grouping of samples in single-cell sequencing dataset GSE168652. **(B)**. The angiogenesis scores for per-cell of GSE168652. **(C)**. Distribution of high and low angiogenesis score cell groups. **(D)**. Samples were clustered by KNN clustering algorithm. **(E)**. Localization of six type of major cells in the sample. **(F)**. Angiogenesis score for each cervical cancer patient from TCGA-CESC cohort. **(G,H)**. Blue, greenyellow, magenta, purple, red, turquoise, pink, and yellow modules were closely related to angiogenesis-related scores.

First, quality control for the single-cell sequencing data was performed to observe whether there was a batch effect (Supplementary Figures S6A, B). As shown in Figure 2A, there is no significant batch effect was observed in GSE168652, which could be used for subsequent analysis. 4,421 cells in adjacent tissues and 2,882 cells in tumor were obtained after screening. Based on median angiogenesis-related gene proportion, all cells were classified into high and low-AGS cells group respectively (Figures 2B, C). Through performing the k-Nearest Neighbor (KNN) clustering algorithm, we divided the samples into 25 clusters (Figure 2D), and classified these clusters into six major cell types according to surface marker genes (Supplementary Table S1): tumor/epithelial cells, smooth muscle cells, macrophages, fibroblasts, lymphocytes, and endothelial cells (Figure 2E). Moreover, the localized difference of cell marker genes was displayed in GSE168652 (Supplementary Figure S1). Meanwhile, in TCGA-CESC cohort, each cervical cancer patient was scored for the AG gene-set (Figure 2F). Then the weighted co-expression network analysis (WGCNA) based on angiogenesis phenotype was performed (Figure 2G). The soft threshold was set to 20 (Supplementary Figure S6C), with a minimum module gene count of 60 and deepsplit of 2. Then we merged modules with similarity was less than 0.1 and combined the remaining modules into another class (Supplementary Figure S6D). A total of 11 non-gray modules (Figure 2H) were obtained. Module based on AGS were included (*p*-value < 0.05): blue, greenyellow, magenta, purple, red, turquoise, pink, and yellow. We included all genes in the module based on AGS (*p*-value < 0.05) as phenotypically related genes for the follow-up study.

#### Construction and validation the prognostic model based on angiogenesis-related genes

**FIGURE 3:**
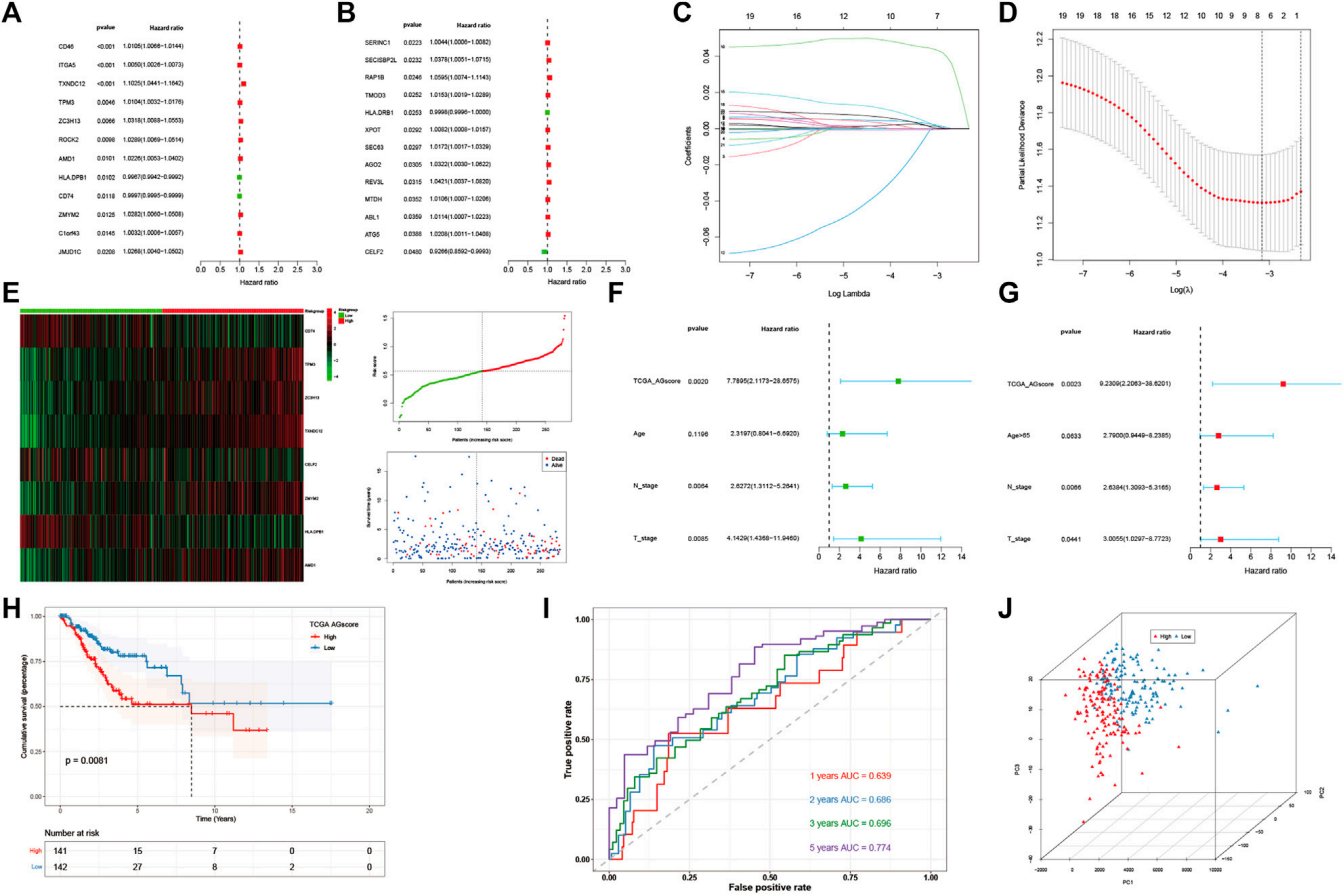
Construction and evaluation of angiogenesis-related prognostic model. **(A,B)**. 25 AG genes associated with prognostic screened by univariate cox analysis. **(C,D)**. Distribution of lasso regression curves. **(E)** Heatmap and point plots for assessing the risk distribution of patients of TCGA-CESC cohort in high- and low-AGS groups. **(F)** Univariate cox analysis of AG modeling scores and clinical characteristics. **(G)**. Multivariate cox analysis of AG modeling scores and clinical characteristics. **(H)**. Survival analysis for TCGA-CESC cohort in high- and low-AGS groups. **(I)**. ROC curves of TCGA-CESC cohort. **(J)**. PCA analysis in TCGA-CESC cohort.

Then 184 genes were obtained by taking the intersection of WGCNA angiogenic module genes and differential gene (DEGs) of GSE168652. Further, a model of 25 genes was set by univariate COX regression analysis (Figures 3A, B). The lasso regression analysis was then performed, and the model was optimized when the number of genes was 8 (Figures 3C, D). Eight genes were: *CD74*, *TPM3*, *ZC3H13*, *TXNDC12*, *CELF2*, *ZMYM2*, *HLA-DPB1*, *AMD1*. The prognostic model was constructed as follows: Angiogenesis-Score = CD74*(−1.687253736851253e-5) + TPM3*0.002624694772647254 + ZC3H13*0.0014952296381226275 + TXNDC12*0.044584570481779856 + CELF2*(−8.09890285266551e-4) + ZMYM2*0.0030029866624357044 + HLA-DPB1*(−6.522321139043119e-4) + AMD1*0.0011052396498335048. By the scores of the prognostic model, we divided total patients in TCGA-CESC cohort into high and low-AGS groups (Figure 3E), and used GSE44001 dataset in GEO website as external validation. Then we analyzed the prognosis of total TCGA patients (Figure3H) and GSE44001 dataset (Supplementary Figure S9I) patients. The results showed that the patients in the high-AGS group had a worse prognosis compared to low-AGS groups. In addition, we constructed ROC curves to explore the accuracy of the model in evaluation of the prognosis. The AUC values at 1, 2, 3, and 5 years for patients with cervical cancer in the TCGA-CESC cohort were 0.639, 0.686, 0.696, and 0.774, respectively (Figure 3I). At the same time, the AUC values at 1, 2, 3, and 5 years for the GSE44001 patient data were validated in Supplementary Figure S9J. Meanwhile, we divided the 283 samples of TCGA-CESC patients into train and test sets according to the random sampling principle in the ratio of 8.5 to 1.5 (241:42). Then we analyzed the survival curve and ROC curve of TCGA train set (Supplementary Figures S9A, B, C) and test set (Supplementary Figures S9D, E, F). The AUC values at 1, 2, 3, and 5 years in TCGA train and test were 0.594, 0.650, 0.647, 0.742, and 0.854, 0.906, 0.962, 1.000 respectively. Finally, we used PCA analysis (3D) in the high and low-AGS groups, and found that the model could group patients well in the two groups (Figure 3J; Supplementary Figures S9G, H), and also displayed the two-dimensional diagram of PCA analysis (Supplementary Figure S6E). Meanwhile, we included clinical characteristics of TCGA-CESC patients and scores of the prognostic model for univariate (Figure 3F) and multivariate cox analyses (Figure 3G). We classified patients according to model score, age <65 or >65 years, T1-2 stage for early stage, T3-4 for late stage, and presence or absence of lymph node metastasis, respectively. The results showed that model score, N stage, and T stage were risk factors for univariate regression. The multivariate cox results likewise indicated that model score, N stage, and T stage were independent risk factors. In addition, we evaluated the accuracy of the model by ridge regression (Supplementary Figure S6H) and lasso regression (Supplementary Figure S6I) in the IOBR algorithm. AUC value of 0.92 in the training set and an AUC value of 0.76 in the validation set by ridge regression analysis. The AUC of the training set was 0.93 and the AUC of the test set was 0.79 for lasso regression. Also, when the outcome event was set as survival status by extracting 70% of the data as the training set, we found that the AUC of the training set was 0.68 and the AUC of the test set was 0.63 by ridge regression (Supplementary Figure S6J). It is showed that the model has good prediction ability.

#### Immune infiltration analysis and mutation landscape and proteomic analysis

**FIGURE 4:**
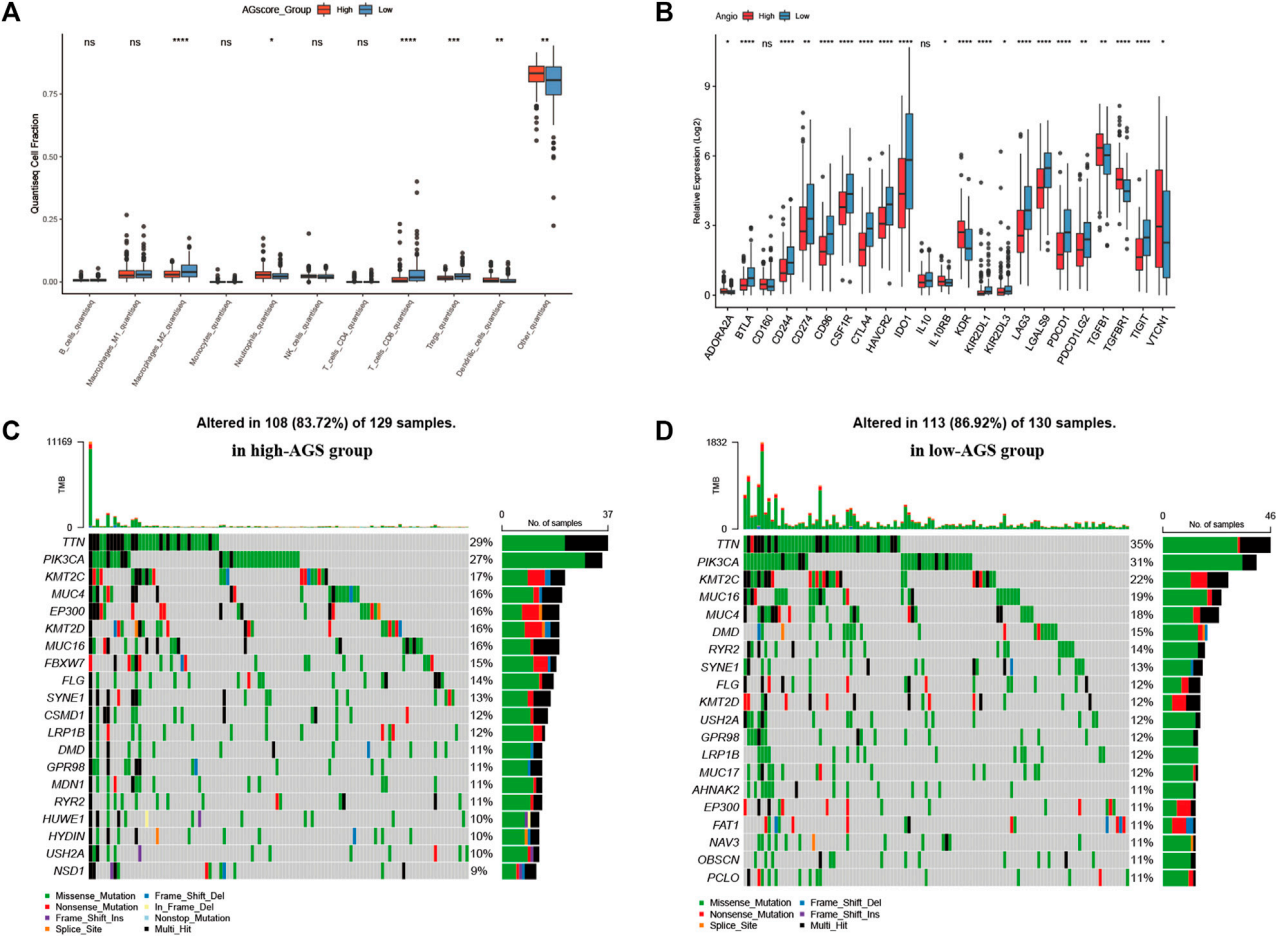
Immune infiltration, tumor mutational burden, and proteomic analysis. **(A).** Expression of 10 types of immune cells in high-AGS group and low-AGS group. **(B)**. Differential expression of immune checkpoint related genes in high-AGS group and low-AGS group. **(C)**. Mutation landscape in high-AGS group of TCGA cohort. **(D)**. Mutation landscape in low-AGS group of TCGA cohort. (* < 0.05, ** < 0.01, *** < 0.001).

After establishing a prognostic model, patients between high or low AGS subgroups had different prognosis. Thus, we began to investigate the different immune infiltration between patients in the AGS groups in the TCGA-CESC cohort. Six algorithms were performed to evaluate difference (Supplementary Figure S7). It was showed that there was higher immune infiltration rate in the low-AGS group in both six types of algorithms. Among them, the Xcell algorithm obtained the most significant comparison in two groups. Next, we analyzed the difference in expression among 10 types of immune cells in the two subgroups (Figure 4A). M2 macrophages, CD8 T-cell, and regulatory T-cell had a higher percentage of immune infiltration in the low-AGS group, while neutrophils and dendritic cells had a higher percentage of immune infiltration in the high-AGS group. Then we validated the differences in neutrophil infiltration in cervical cancer tissues by IHC experiments. The results showed that a significant positive correlation between the high infiltration of neutrophil and high angiogenic expression (Figure 8). Also, we analyzed the differences in the expression of immune checkpoint genes in the two groups (Figure 4B). The expression of most immune checkpoint genes was significantly different between the high and low AGS groups. *IL10RB*, *KDR*, *TGFB1*, *TGFBR1*, and *VTCN1* were upregulated in the high AGS group compared with the low AGS group; the other genes including *CD274* and *CTLA4* were down-regulated in the high AGS group. Meanwhile, we analyzed significantly different expression of gene-markers of immune cells in immunotherapy. The results showed that there were less immune infiltrating cell such as resting dendritic cells (iDC) in the high AGS group, while more immune infiltrating cell like activated dendritic cells (aDC) in the AGS group (Supplementary Figure S2). Moreover, we analyzed the expression of common marker-genes in CD8 effector T-cell. All marker-genes were downregulated in the high AGS group. Then, we analyzed the expression of common marker-genes for targets of immunotherapy: DNA breakage and repair (DDR) and immune checkpoint genes in IOBR algorithm (Supplementary Figure S3). The results showed that the most majority of DDR genes were upregulated in the high AGS group compared to the low AGS group, while immune checkpoint genes were downregulated in the high AGS group. Then we analyzed the differences in tumor mutational genes within and between the two groups. We found that mutation landscape in two group (Figure 4C). As it was displayed, the top five mutated genes were namely *TTN*, *PIK3CA*, *KMT2C*, *MUC4*, and *EP300* in the high AGS group, while the top five mutated genes were *TTN*, *PIK3CA*, *KMT2C*, *MUC16*, *MUC4* and in the low AGS group (Figure 4D). Next, we evaluated differentially mutated genes with significance *p* < 0.05, OR<1 between the two groups (Supplementary Figure S8), and the top 5 ranked genes were *SIPA1L3*, *RBFOX1*, *TTC28*, *NLRP4*, *CNTRL*. Finally, we performed the proteomic analysis by RPPA dataset, and showed their differential proteins such as CASPASE7CLEAVEDD198, EEF2K with volcano plots between subgroups (Supplementary Figure S6G).

#### Cell localization of modeling genes in single-cell sequencing dataset

**FIGURE 5:**
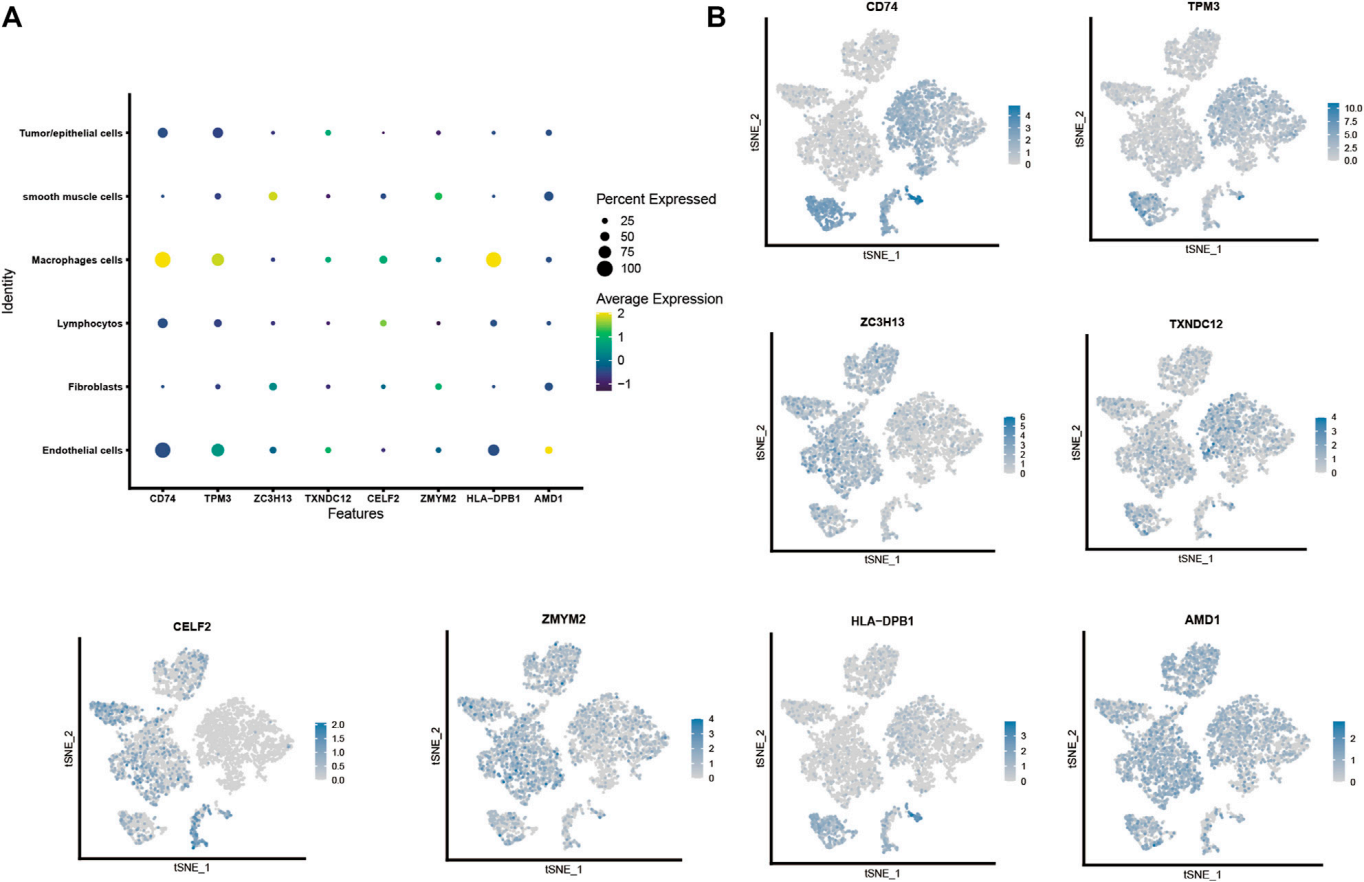
Cell localization of eight modeling genes in the single-cell sequencing dataset. **(A,B)**. Distribution and expression of eight prognostic modeling genes in six type of cells.

We plotted the gene expression of the eight key genes screened by the lasso regression model in the single-cell sequencing data to observe their main distribution. Firstly, we observed the overall distribution and expression of the eight genes (Figure 5A), followed by the distribution of each gene in cells (Figure 5B): *CD74* was mainly expressed in macrophages. *TPM3* was mainly expressed in macrophages, lymphocytes, and endothelial cells. *ZC3H13* was mainly expressed in smooth muscle cells, fibroblasts, and *TXNDC12* was mainly expressed in tumor/epithelial cells, macrophages, and endothelial cells. *CELF2* was mainly expressed in macrophages, lymphocytes, and *ZMYM2* was mainly expressed in smooth muscle cells and fibroblasts. *HLA-DPB1* was mainly expressed in macrophages, and *AMD1* was mainly expressed in smooth muscle cells, fibroblasts, and endothelial cells.

#### Construction of nomogram and decision curve analysis

**FIGURE 6:**
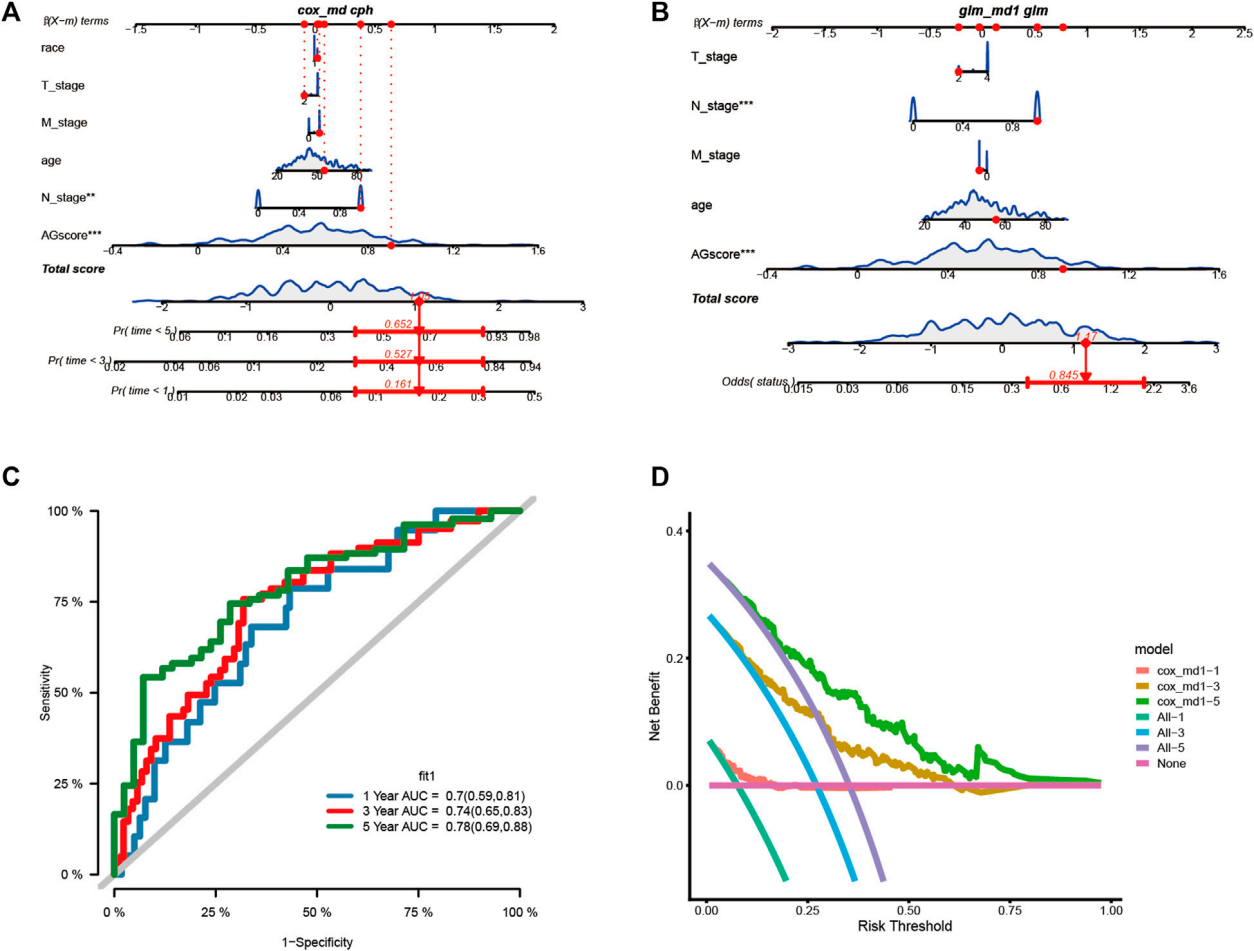
Construction the nomogram and decision curve analysis. **(A)**. Construction nomogram to predict 1-, 3-, and 5-year clinical outcome by cox regression. **(B)**. Construction nomogram to predict status of patients by logistic regression. **(C)**. ROC curve of the nomogram. **(D)**. Decision curve analysis. (* < 0.05, ** < 0.01, *** < 0.001)

We assessed the clinical outcome of patients by constructing a nomogram based on AGS values compared with other clinical characteristics. According to clinical characteristics such as ethnicity, TNM stage, age, and AGS values, scoring cumulatively by cox regression method (Figure 6A), the mortality rate of the patient in 1, 3 and 5 years was estimated to be 0.161, 0.527, and 0.652 respectively. At the same time**,** up to the time of follow-up of TCGA-CESC cohort, the rate of status outcome was estimated to be 0.845 by logistic regression methods (Figure 6B). The area under the ROC curve for 1, 3, and 5 years was 0.70, 0.74 and 0.78, respectively (Figure 6C). It indicates that the nomogram had good predictive ability. From the nomogram, the results showed that the AGS values had significant effect on the prognosis. Meanwhile, we used decision curve analysis (Figure 6D). The results suggested that the nomogram with mortality rates of 1, 3, and 5 had significant optimization compared with those without constructing nomogram, indicating that the nomogram has good predictive ability on prognosis.

#### Selection and validation of prognosis-related signature genes

**FIGURE 7:**
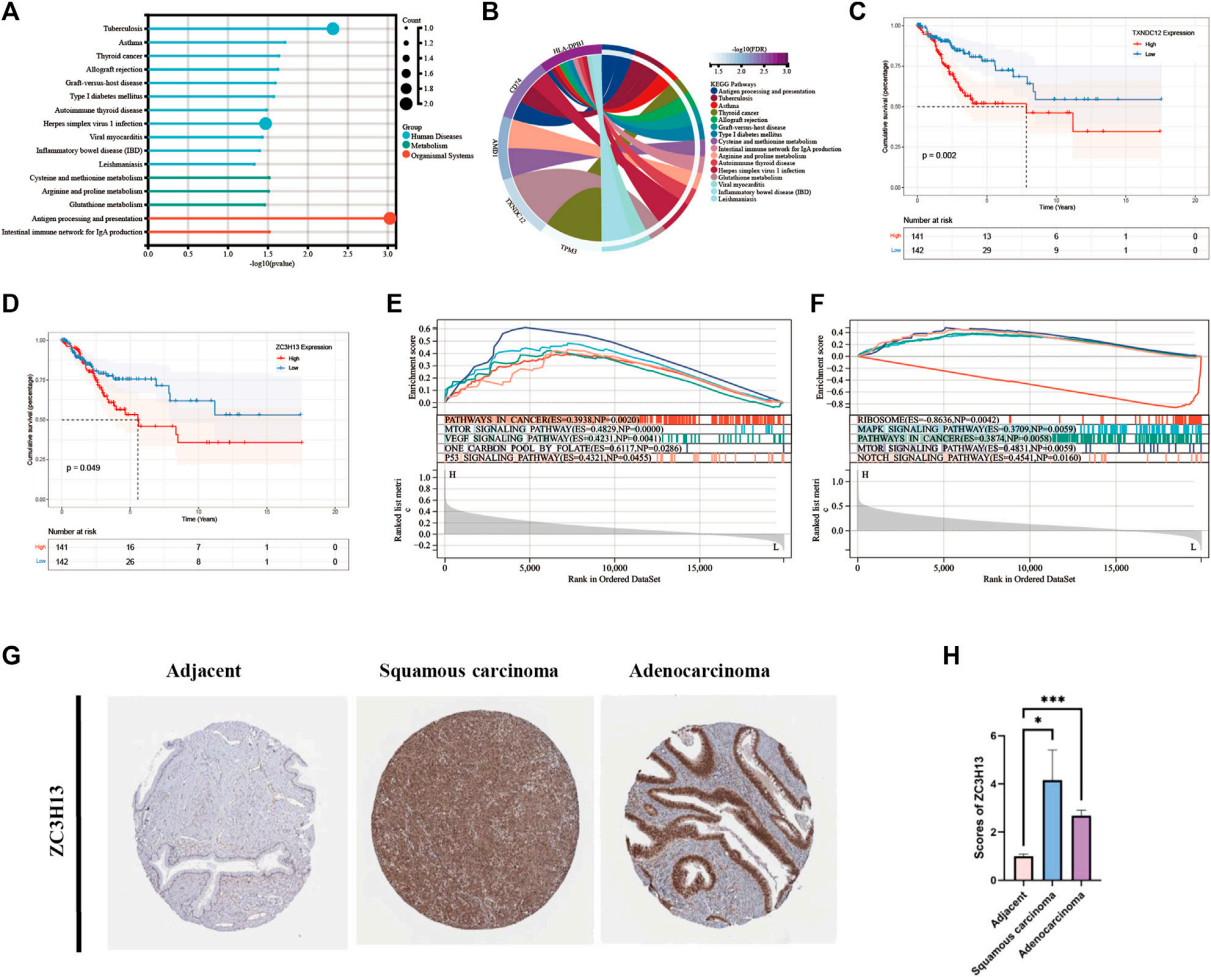
KEGG and GSEA analysis and Survival analysis of modeling genes and validation of ZC3H13 in CESC tissues. **(A)**. Histogram of KEGG pathway. **(B)**. Circle plot of KEGG pathway of modeling genes. **(C)**. Survival curve of *TXNDC12*. **(D)**. Survival curve of *ZC3H13*. **(E)**. GSEA pathway analysis of *TXNDC12*. **(F)** GSEA pathway analysis of *ZC3H13*.**(G,H).** Quantification and comparison of protein expression of ZC3H13 by IHC. (* < 0.05, ** < 0.01, *** < 0.001).

Eight modeling genes were divided into high and low expression groups according to median values. The results showed that high expression of *TXNDC12* and *ZC3H13* had a significantly worse prognosis (Figures 7C, D). Further, we ranked the eight genes according to their HR value in the prognostic model (Supplementary Table S2), and observed lasso regression coefficients of the model (Supplementary Table S3). It suggested that the highest HR value and coefficient of *TXNDC12* could have association with cervical carcinogenesis. We also evaluated the role of *TXNDC12* (Supplementary Figure S4) and *ZC3H13* (Supplementary Figure S5) in the immune microenvironment. The microenvironment-score and immune-score were significantly downregulated by high expression of *TXNDC12* and *ZC3H13*. Therefore, we performed RT-qPCR experiment. The results showed *TXNDC12* and *ZC3H13* were highly expressed in tumor cells compared to normal cells. This further validated our speculation.

#### Comparison of modeling genes related multiple pathways and validation of prognosis-related signature genes in CESC tissues

We performed analysis of the enrichment of KEGG pathways (Supplementary Table S4). The modeling genes are significant enrichment in the pathways of human disease such as tuberculosis, herpes simplex virus one infection, and in the pathways of organizational system such as antigen processing and presentation (Figure 7A). As displayed in Figure 7B, *TXNDC12* was mainly enriched in glutathione metabolism pathway; *AMD1* was mainly involved in cysteine and methionine metabolism, arginine and proline metabolism; *TPM3* was mainly involved in cardiac muscle contraction, dilated cardiomyopathy, and dilated cardiomyopathy; *CD74* is involved in antigen processing and presentation pathway; *HLA-DPB1* is involved in viral myocarditis pathway. Then, we performed GSEA analysis of *TXNDC12* and *ZC3H13* to identify 5 enrichment pathways of significant significance. It was found that high expression of *TXNDC12* was significantly associated with one carbon pool by folate pathway, mTOR signaling pathway, p53 signaling pathway, VEGF signaling pathway, pathways in cancer (Figure 7E). High *ZC3H13* expression was associated with low expression of the RIBOSOME, positive correlation of the MAPK signaling pathway, pathways in cancer and mTOR signaling pathway, NOTCH signaling pathway (Figure 7F). We further explored the protein expression of the prognostic modeling genes ZC3H13 in adjacent and CESC tissues. As shown in Figures 7G, H, the results of immunohistochemistry (IHC) from HPA database showed that the expression levels of ZC3H13 were significantly higher in cervical squamous cell carcinoma or adenocarcinoma than in adjacent normal tissues. And then, indicating that prognosis-related signature genes may play an important role in cervical cancer progression.

#### The expression of TXNDC12 is upregulated in cervical cancer tissues and positively correlates with angiogenesis

**FIGURE 8:**
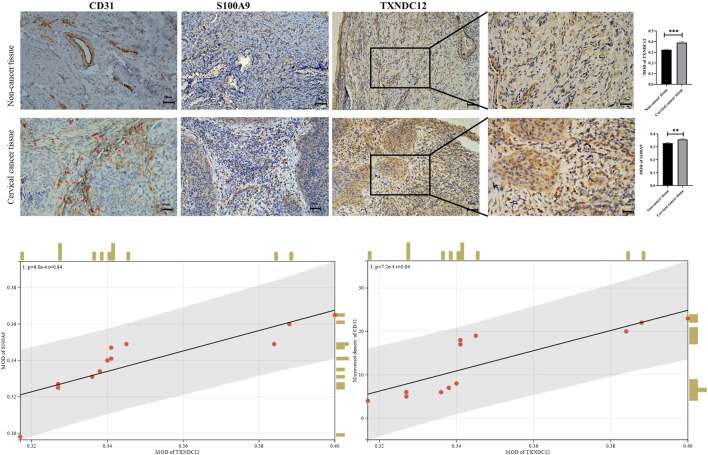
Immunohistochemical staining and correlation analysis of CD31, S100A9, TXNDC12.

We also analyzed the expression of TXNDC12, CD31 (neovascularization marker gene), and S100A9 (neutrophil marker gene) by IHC experiments in cervical cancer tissues (Figure 8). The results showed that the expression of high TXNDC12 was significantly and positively correlated with the expression of CD31, S100A9. High TXNDC12 expression may be positively associated with the presence of higher neovascularization and infiltration of neutrophils in patients.

#### Functional validation of TXNDC12 in cervical cancer

**FIGURE 9:**
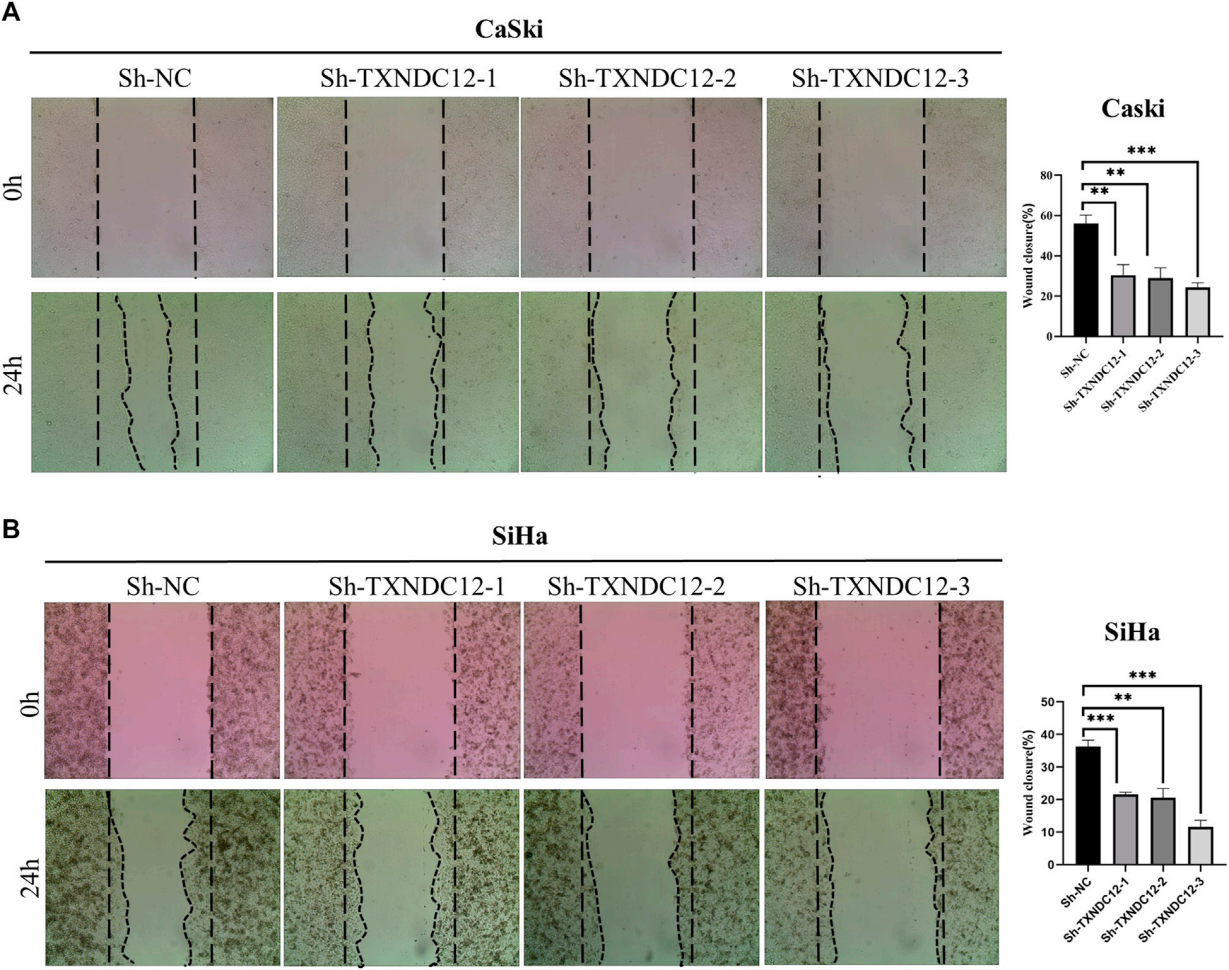
Scratch wound healing assay with CaSki cell line **(A)** and SiHa cell line **(B)**. (* < 0.05, ** < 0.01, *** < 0.001).

**FIGURE 10:**
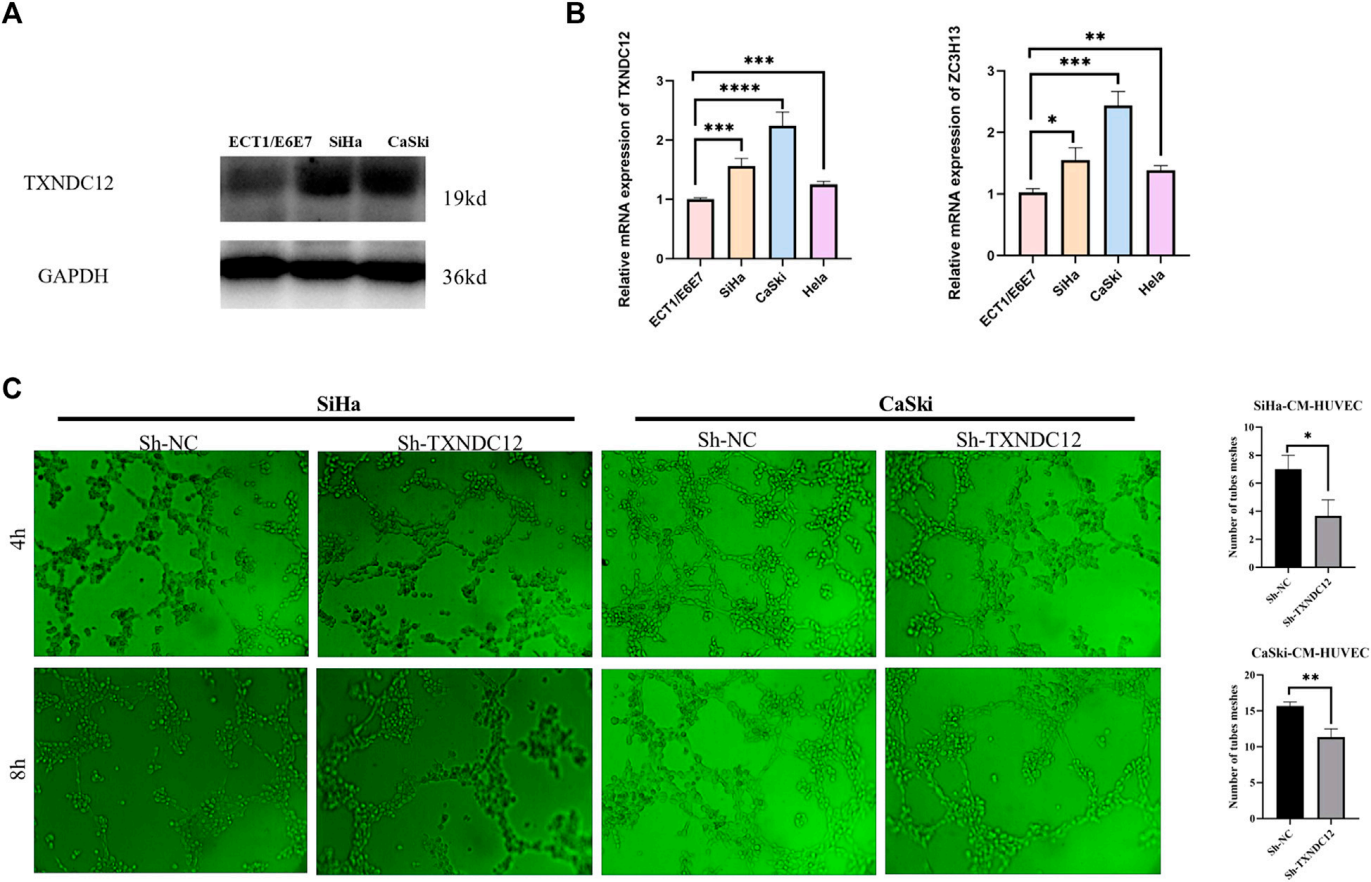
TXNDC12 expression and tubule formation assay. **(A,B)** Western blotting and qPCR to verify the expression of TXNDC12. **(C)**. Tubule formation assay of endothelial cells. (* < 0.05, ** < 0.01, *** < 0.001).

To verify the effect of TXNDC12 on tumor progression and angiogenesis in cervical cancer, we performed western blotting (Figure 10A), scratch wound healing assay (Figures 9A, B), tubule formation assay (Figure 10C). The results showed that *TXNDC12* was highly expressed in cervical cancer cell lines (Figure 10B). Meanwhile, it was found that the migratory ability of SiHa and CaSki cells and the ability of endothelial cell tubule formation were significantly inhibited in the sh-*TXNDC12* group compared with the control group, and the differences were statistically significant.

#### Potential small molecule drug prediction and drug sensitivity analysis of model genes

**FIGURE 11:**
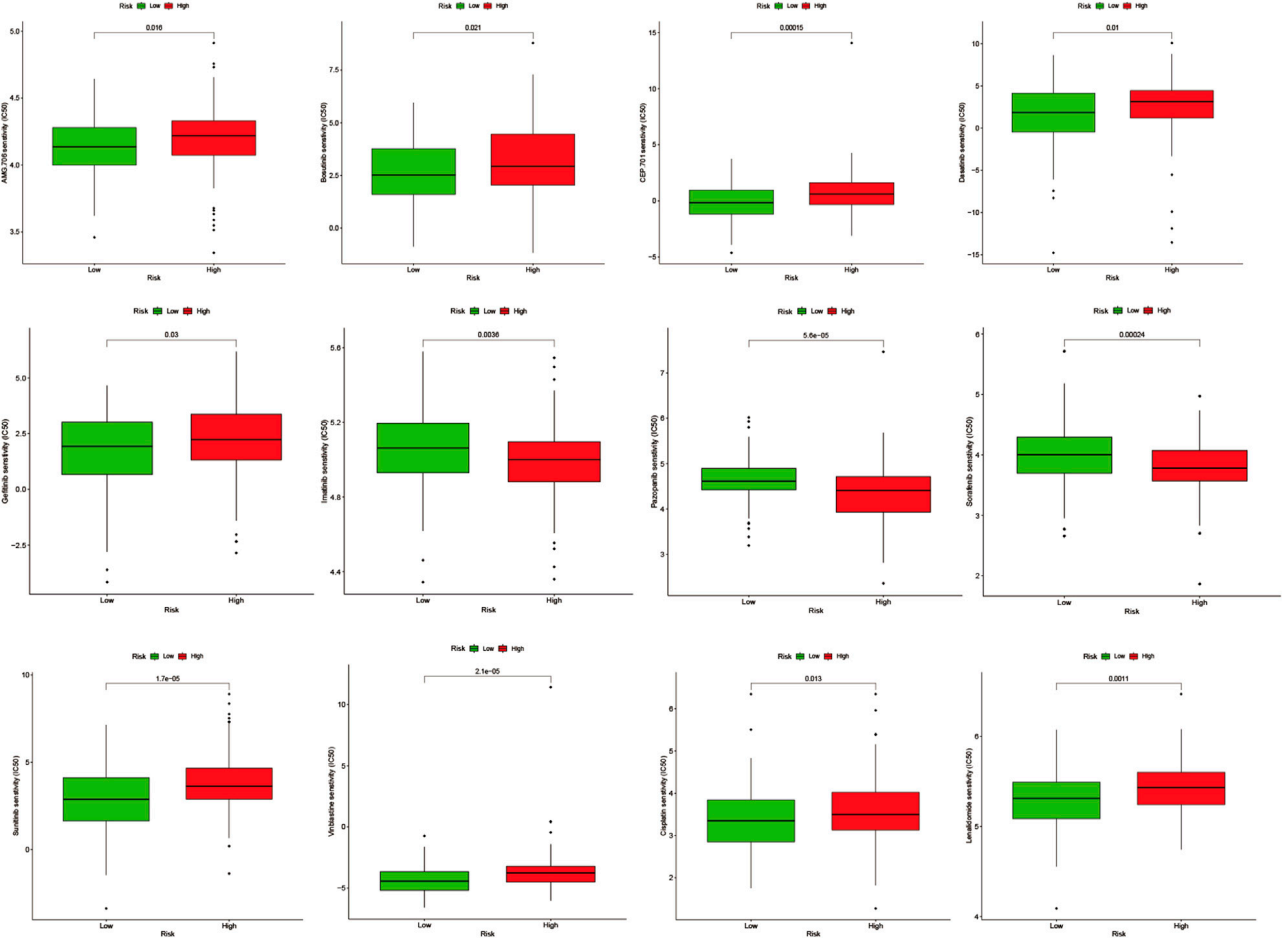
Drug sensitivity analysis in the high and low AGS group.

We performed drug prediction of potential small molecules in the DSigDB database for model genes, and we found Penconazole CTD 00003093, bisacodyl MCF7 UP, Nickelous acetate CTD 00003684, Zinc sulfate CTD 00007264, staurosporine MCF7 DOWN, VALPROIC ACID CTD 00006977 would be effective small molecule drug (Supplementary Table S5). Meanwhile, we searched the GDSC database by IC50 analysis (Figure 11) for anti-angiogenesis-related VEGFR receptor inhibitors as well as tyrosinase inhibitors and immunomodulators with significant differences between high- and low-AGS groups. The results suggest that for patients in the low-AGS group, the efficacy of AMG.706, Bosutinib, and Lenalidomide may be superior to that in the high-AGS group while Imatinib, Pazopanib, and Sorafenib may have better efficacy in the high AGS group.

## Discussion

In this study, a new prognostic model in cervical cancer was developed. The model was based on eight AG genes and had independent prognostic significance for patients with cervical cancer. The prediction accuracy of the model was validated in the internal training set, test set, and external validation set. In addition, we explored the differences in immune infiltration, tumor mutational burden (TMB), clinical outcome and drug sensitivity between high and low-AGS groups under the model, and identified two key prognostic genes from them. *TXNDC12* was found to have the ability to promote cell migration in cervical cancer cell line and tubule formation in endothelial cells, which can help to give new directions for the treatment of patients with recurrent or refractory cervical cancer in clinic.

Our data were obtained from TCGA, GEO and other databases, and we used a combined multi-omics approach and multi-dimensional study to explore the complex molecular mechanisms of tumor angiogenesis. Single-cell RNA sequencing (scRNA-seq) measures gene expression at the single-cell level, which can reveal complex and rare cell populations thereby better revealing regulatory relationships between genes (Hwang et al., 2018) with higher resolution of cellular differences (Gao, 2018). Heterogeneity in tumors and their microenvironment (Papalexi and Satija, 2018) was revealed in breast cancer (Ding et al., 2020; Yuan et al., 2021), melanoma (Tirosh et al., 2016), pancreatic tumors (Zhou et al., 2021), etc. TCGA and GEO public database has mRNA sequencing data for a large number of samples, which is more convincing when combined with single cell sequencing database to build a model analysis (Zoabi and Shomron, 2021). In this paper, we construct a model based on the AGS. We explored the set of AG genes and looked at their immune infiltration and tumor mutational burden. This modeling approach that divides the study population into high and low groups takes into account the overall impact of AG genes and is more comprehensive in assessing differences in prognosis. First, we started from the data of scRNA-seq to find the relevant differential genes in the high and low AGS-cell groups, and further applied the TCGA data for cox regression and lasso regression analysis to model the prognosis of AG genes, and then we found that significant differences in the survival rates of high and low AGS-patients group. The AUC values exceeded 0.742 and 1in the internal train and test sets respectively, indicating that our model has good accuracy in predicting prognosis. We explored the efficacy of the AG scores with clinically relevant features in predicting clinical outcomes after modeling, with AUC values of 0.70, 0.74, and 0.78 for 1-, 3-, and 5-year survival, and the DCA decision curves similarly illustrated the high predictive accuracy of our model for nomogram.

Eight genes were finally included in our modeling, namely *CD74*, *TPM3*, *ZC3H13*, *TXNDC12*, *CELF2*, *ZMYM2*, *HLA-DBP1*, and *AMD1*. *TXNDC12*, as a member of the thioredoxin superfamily, plays an important role in tumors. In hepatocellular carcinoma (Yuan et al., 2020), *TXNDC12* promotes EMT and metastasis in tumors. And in glioma, high expression of *TXNDC12* predicts the poor prognosis of patients (Wang X. et al., 2021). *ZC3H13*, an m6A methyltransferase, also plays an important role in tumors (Wang et al., 2020; Song et al., 2022). In breast cancer (Gong et al., 2020) and colorectal cancer (Zhu et al., 2019), *ZC3H13* plays a tumor suppressive role, while in hepatocellular carcinoma, *ZC3H13* promotes the malignant behavior of hepatocellular carcinoma cells (Wang Q. et al., 2021). *CD74*, a key molecule involved in antigen presentation, B-cell differentiation and inflammatory signaling, could be considered as a new candidate target and vaccine for tumor immunotherapy to combat tumors (Stein et al., 2007; Borghese and Clanchy, 2011). *TPM3* is reported to cause tumorigenesis (Mano, 2012). Antibodies against *TPM3* are used for the early diagnosis of endometriosis (Greenbaum et al., 2021). *CELF2*, which belongs to the *CELF*/Bruno-like family of RNA-binding proteins, is involved in selective splicing as well as translation and stability control of target mRNAs(Barreau et al., 2006). *ZMYM2*, which is *FGFR1* (fibroblast growth factor receptor 1) is one of the most common chaperones, and patients with *ZMYM2-FGFR1* fusions frequently present with myeloproliferative neoplasms and T-lymphocytic lymphomas (Katoh, 2016; Montenegro-Garreaud et al., 2017). *HLA-DPB1* is strongly associated with lymphomagenesis (Xiong and Zhao, 2019) and is associated with rheumatoid arthritis (Jiang et al., 2018). *AMD1* is an important regulator in stemness of hepatocellular carcinoma cells (Bian et al., 2021).

During the process of angiogenesis, the release of multiple cytokines recruits the aggregation of multiple immune cells thereby creating a tumor immune microenvironment (TIME). In recent years, other approaches such as the application of immune checkpoint inhibitors for cervical cancer have been actively explored while considering the limitations of applying angiogenesis blockers (Minion and Tewari, 2018). It shows that targeting tumor vasculature and immune checkpoint genes are a potential strategy to potentiate cancer immunotherapy (Lee et al., 2020). New challenges in clinical trial design should be considered more for the immune system and its interactions with the vascular system (Solimando et al., 2020; Ribatti et al., 2021). In the current phase III clinical trials for cervical cancer, all trials of combination chemotherapy for cervical cancer have been a combination of angiogenesis inhibitors and ICIs (Kagabu et al., 2020). This indicates that it is necessary to study the relationship between the role of angiogenesis and immune cell infiltration. In cervical cancer, we combined six algorithms for immune infiltration analysis of the phenotype of angiogenesis and found that there was higher immune suppression in the high-AGS group. Meanwhile, the common immune checkpoint genes *CD274* and *CTLA4* were significantly upregulated in the low-AGS group, suggesting to us that patients in the low-AGS group may benefit clinically with immune checkpoint inhibitors, while patients in the high-AGS group may not benefit significantly in terms of efficacy when applying *CD274* and *CTLA4* inhibitors. *IL10RB, KDR, TGFB1 TGFBR1*, and *VTCN1* genes were upregulated in the high-AGS group, and attempts to use inhibitors against these targets were considered, among others. Because of the worse prognosis and the presence of higher immunosuppression in patients in the high AGS group, we are more interested in exploring therapeutic strategies to improve the prognosis of these patients. Therefore, we envision that patients with recurrent or metastatic cervical cancer, among whom are classified in the high AGS-group, are more likely to consider paclitaxel + cisplatin (or carboplatin) + bevacizumab + *IL10RB/KDR/TGFB1/TGFBR1/VTCN1* inhibitors, which may contribute to the improvement of OS PFS, ORR, DOR. Meanwhile, in clinical practice, TMB of patients is strongly associated with immune infiltration and prognosis. We found that TMB were lower in the high-AGS group compared to the low-AGS group, suggesting that the high-AGS group has a lower TMB and lower immune infiltration, thus guiding us to improve the treatment of patients with high AGS.

We also analyzed the clinical implications of the angiogenesis model. Overall survival was significantly shorter in the high-AGS group, compared to the low-AGS group. Therefore, it is urgent to find better therapeutic ideas for patients in the high-AGS group to mitigate relapse and progression in their patients. We also analyzed the different drug sensitivities in the high and low AGS groups. Results showed that patients in high AGS group may be more sensitive to tyrosinase inhibitor drugs: Imatinib, Pazopanib, and Sorafenib. We selected model genes *TXNDC12* and *ZC3H13* with consistent prognostic trends in the high-AGS group for further analysis. We performed tumor microenvironmental scoring of these two genes by IOBR algorithm and found that their immune score and total microenvironmental score were significantly downregulated when the genes were highly expressed, which may suggest that *TXNDC12*, *ZC3H13* are involved in immunosuppression. Meanwhile, we detected that mRNA of key prognostic genes *TXNDC12* and *ZC3H13* were highly expressed in cervical cancer cells by RT-qPCR. *TXNDC12* was involved in various metabolic pathways including glutathione pathway and VEGF pathway by KEGG and GESA analysis. It has been noted that angiogenesis can be inhibited through glutathione metabolic activation (Cui et al., 2019). However, even though the literatures point out the role of *TXNDC12* or *ZC3H13* in tumors, the prognostic impact through angiogenesis and thus involvement in cervical cancer remains unclear. We speculate that *TXNDC12*, *ZC3H13* may have potential as a prognostic marker for patients in the high-AGS group. The results of functional experiments showed that *TXNDC12* had enhanced ability to promote endothelial cell tube-formation while promoting cervical cancer cell migration, so *TXNDC12* is hopeful as a new target in the AGS group for anti-angiogenic therapy, and improve new thinking and research direction for clinical treatment.

In conclusion, the new prognostic model based on AGS may provide new options for the treatment of cervical cancer. However, we only did expression validation at the cellular level, and specific mechanistic studies remain to be explored in anticipation of further validation in animals and humans in the future.

## Data Availability

The datasets presented in this study can be found in online repositories. The names of the repository/repositories and accession number(s) can be found in the article/Supplementary Material.
