# Supplementary figures and images for: Angiogenesis-related gene signatures reveal the prognosis of cervical cancer based on single cell sequencing and co-expression network analysis
Source: Front Cell Dev Biol. 2023 Jan 12;10:1086835. doi: 10.3389/fcell.2022.1086835 (PMC9877352; doi:10.3389/fcell.2022.1086835)

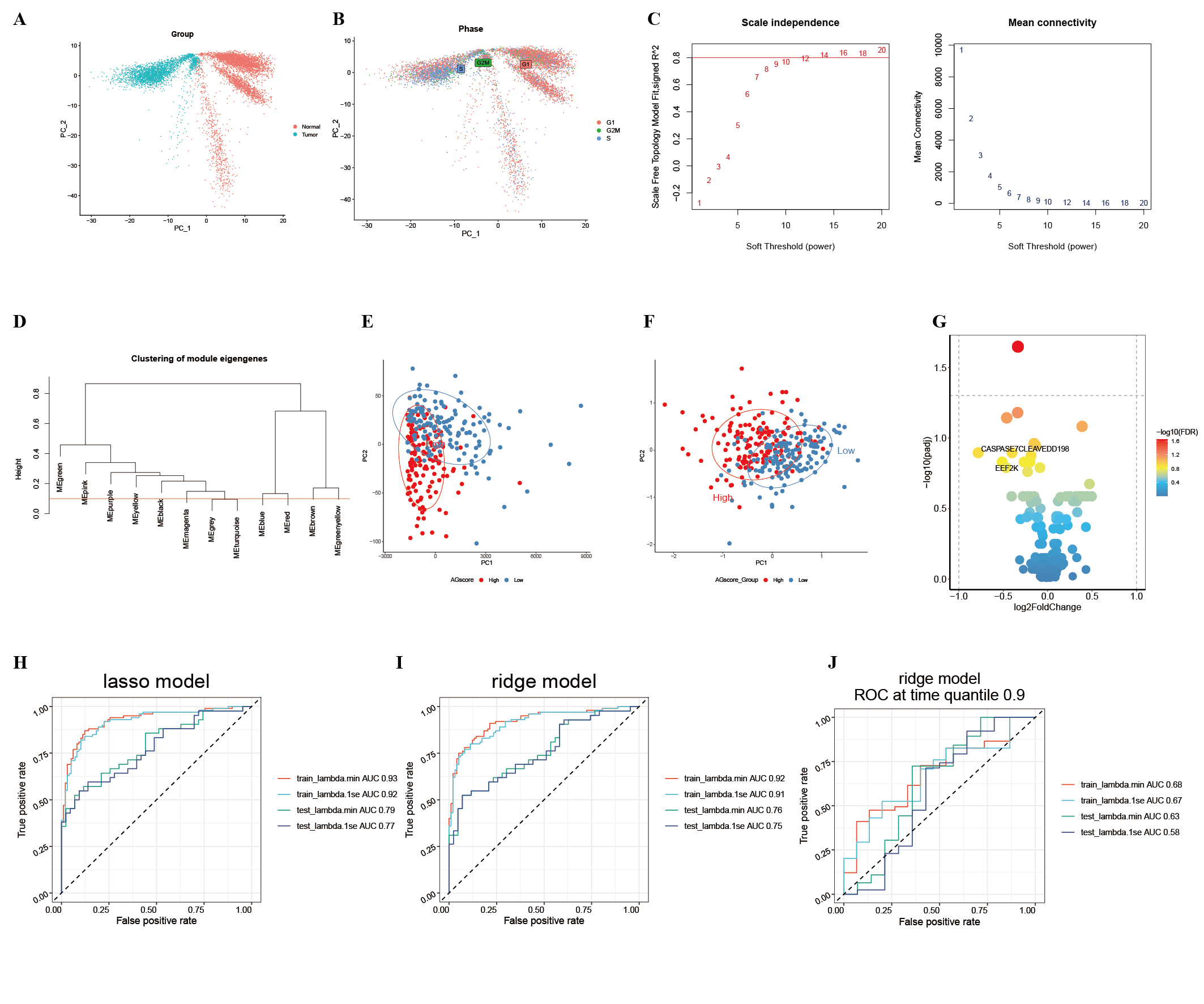

Supplement: Supplementary file 1 [file Image6.tif]

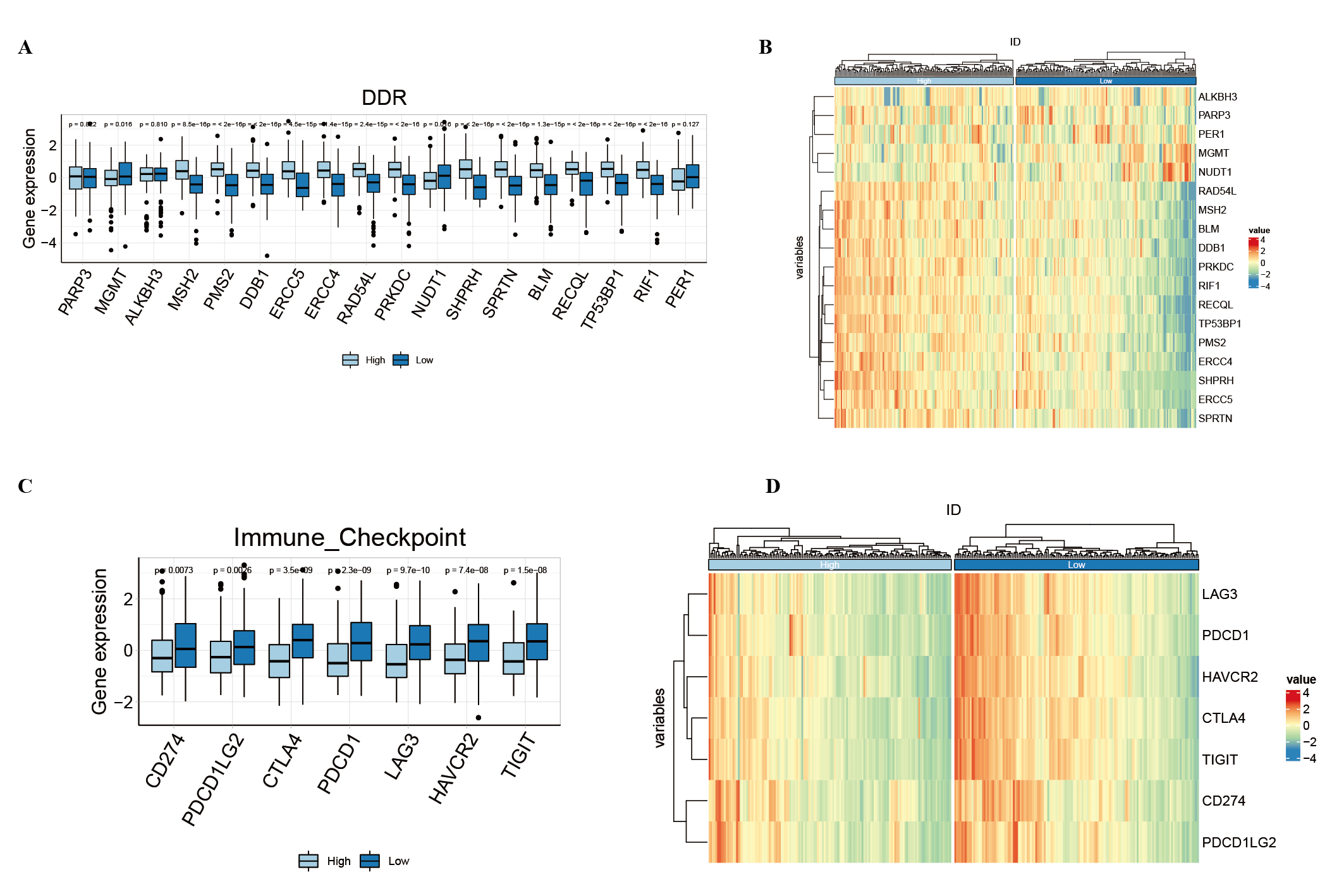

Supplement: Supplementary file 3 [file Image3.tif]

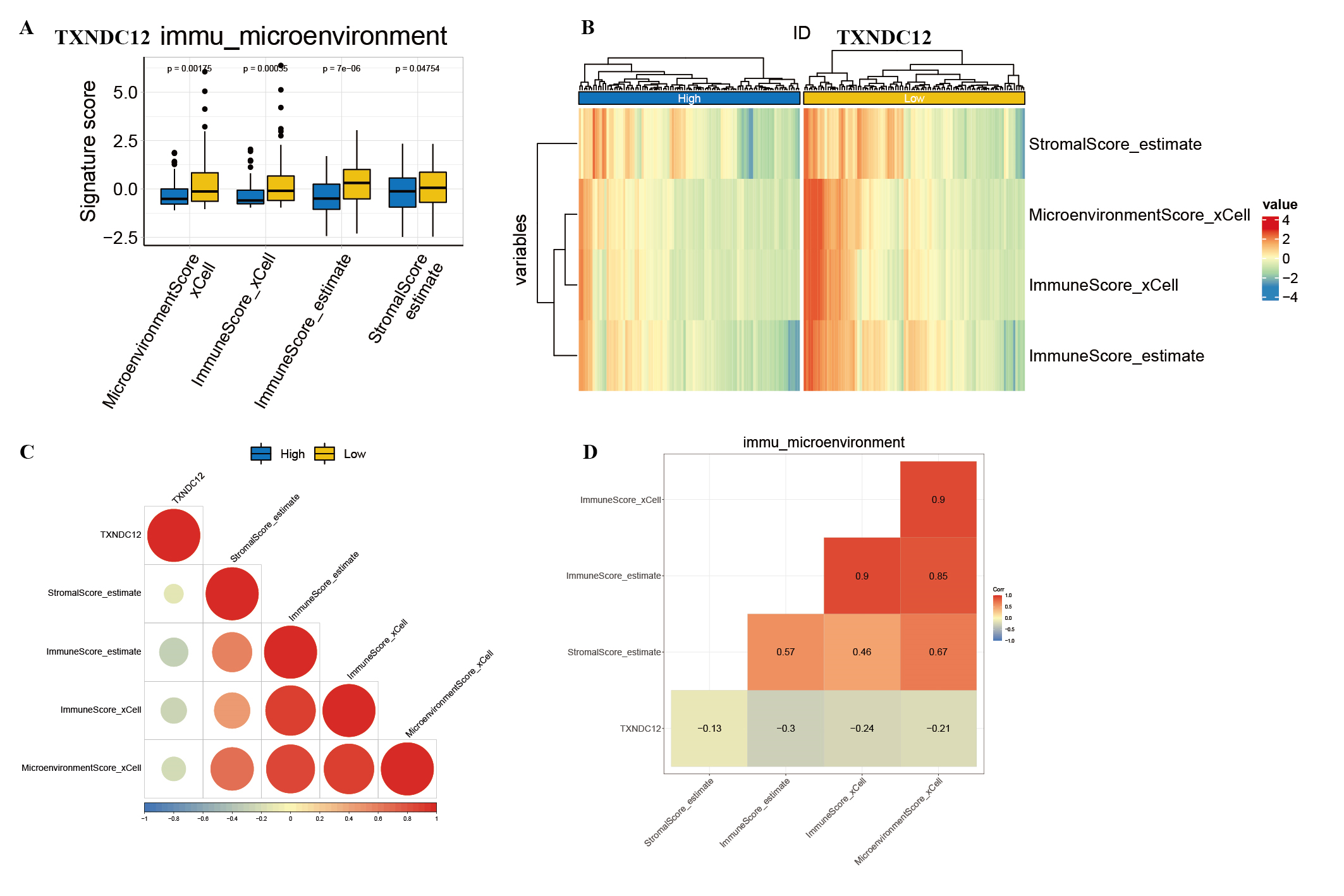

Supplement: Supplementary file 4 [file Image4.tif]

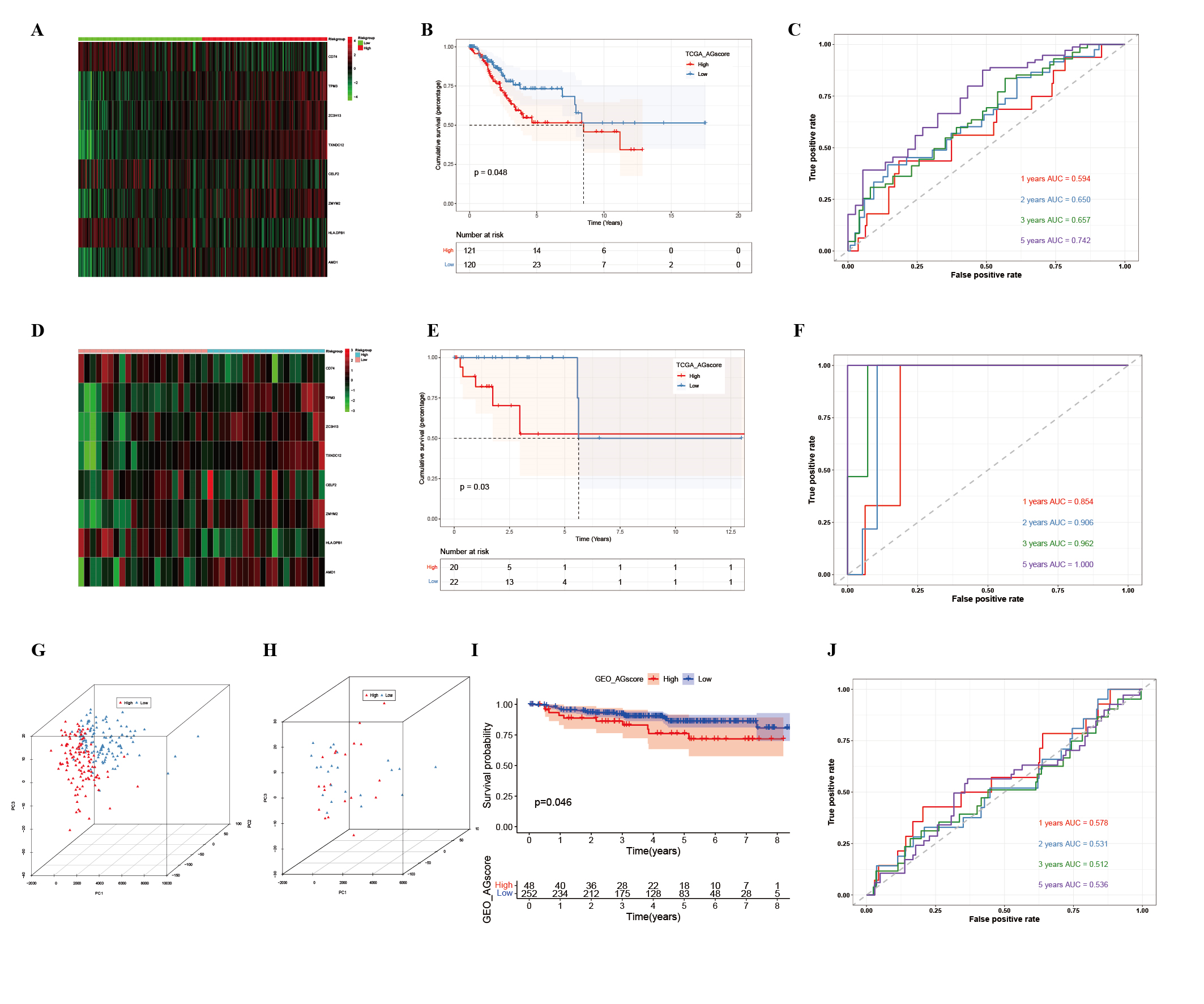

Supplement: Supplementary file 5 [file Image9.tif]

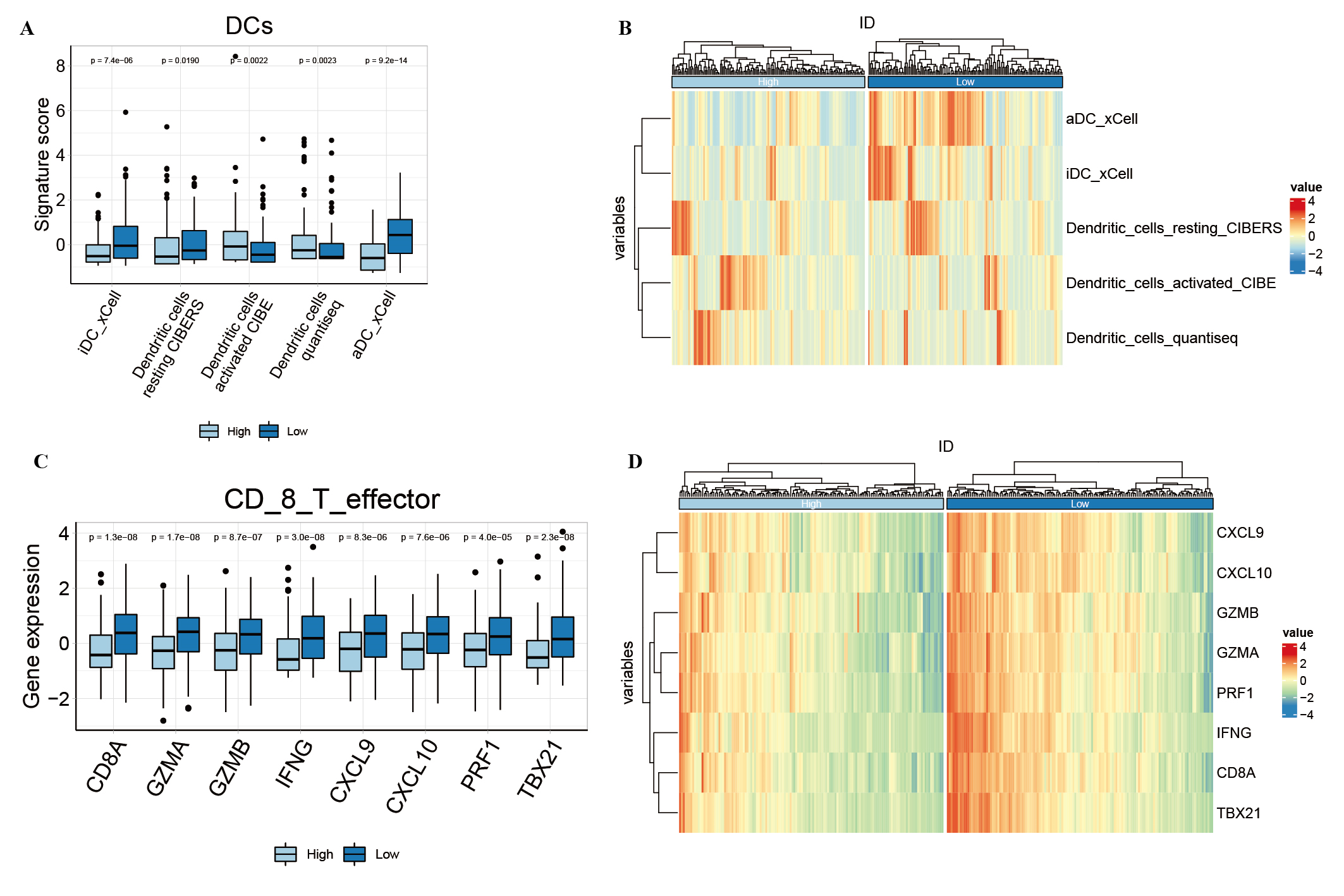

Supplement: Supplementary file 6 [file Image2.tif]

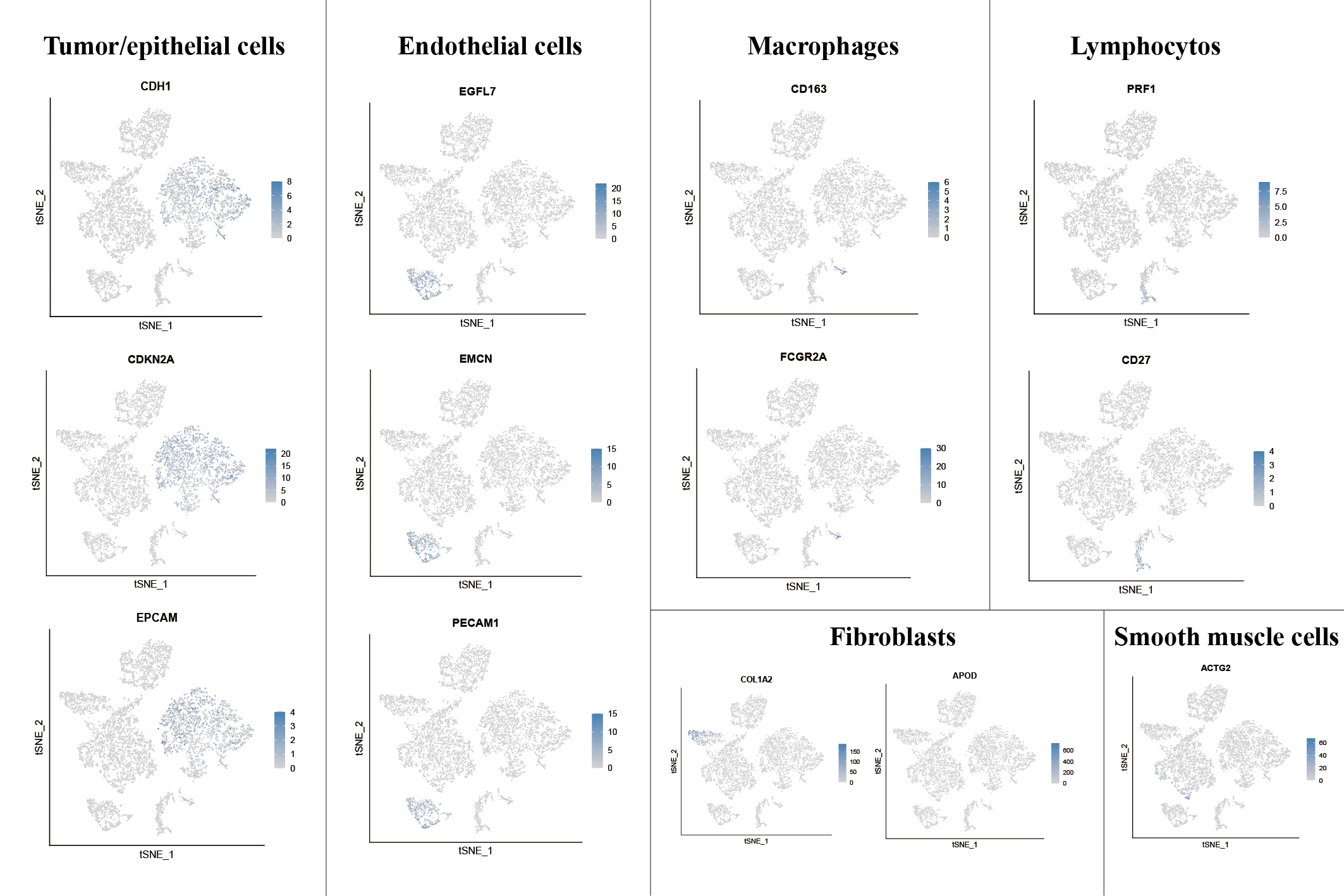

Supplement: Supplementary file 7 [file Image1.tif]

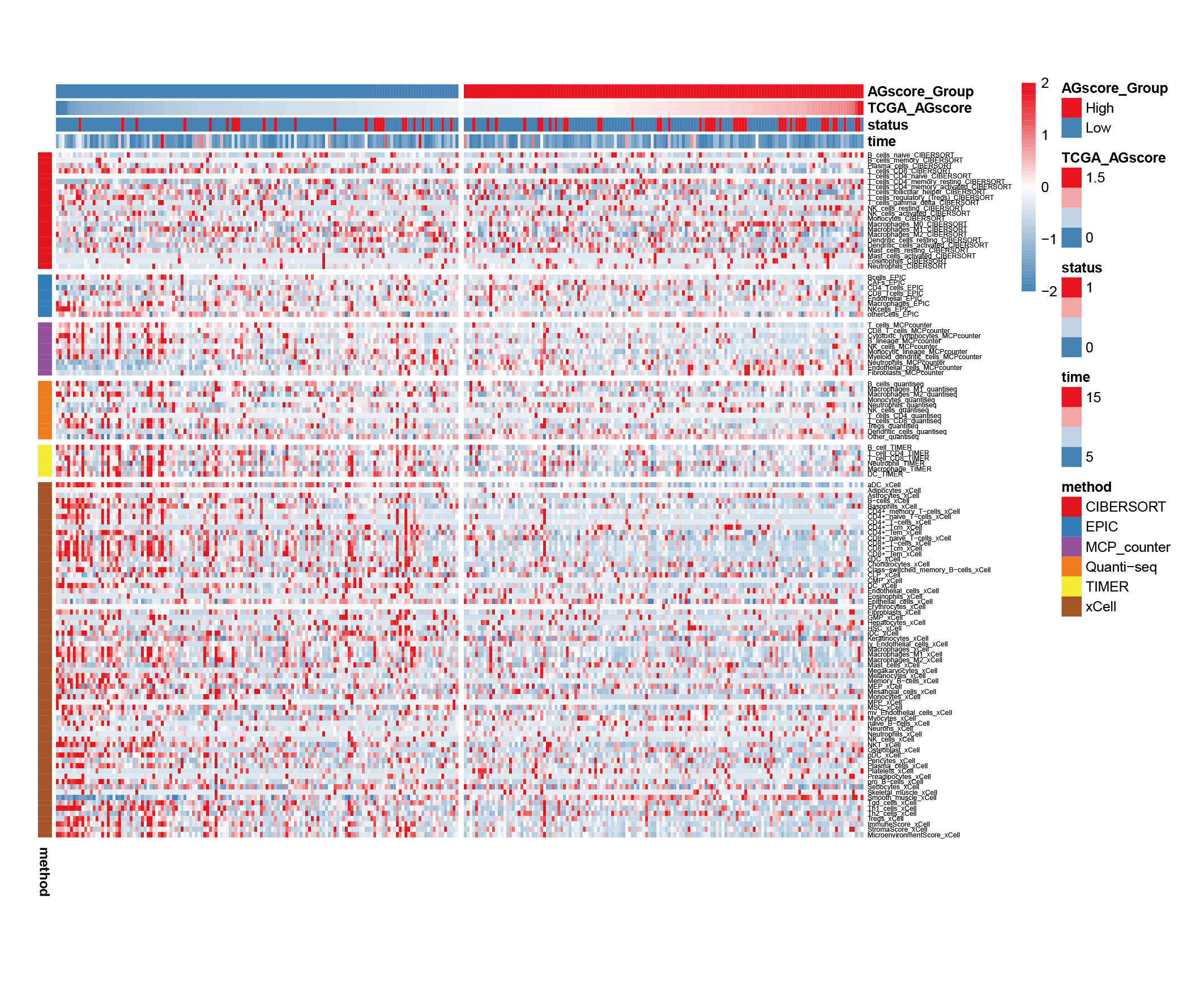

Supplement: Supplementary file 8 [file Image7.tif]

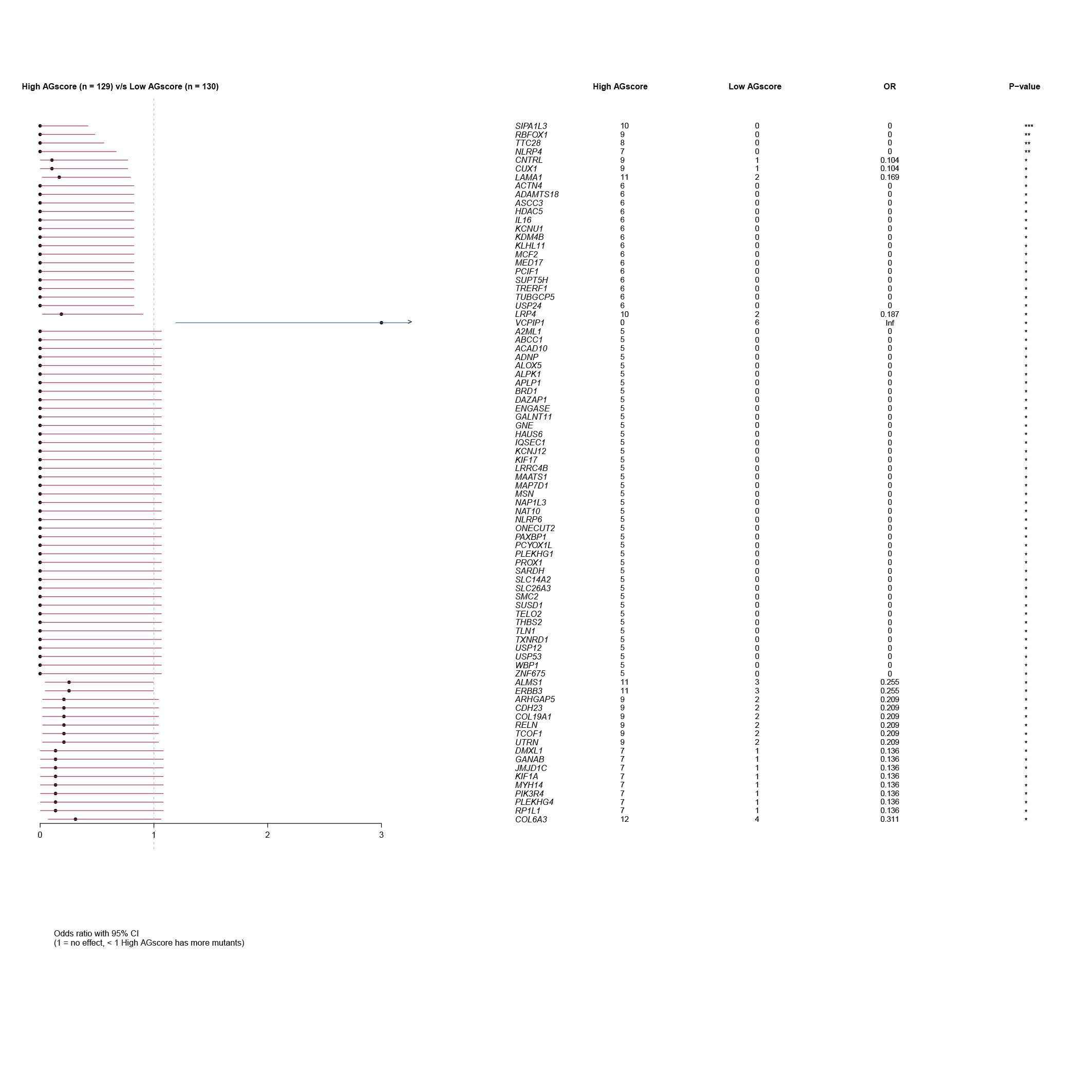

Supplement: Supplementary file 9 [file Image8.tif]

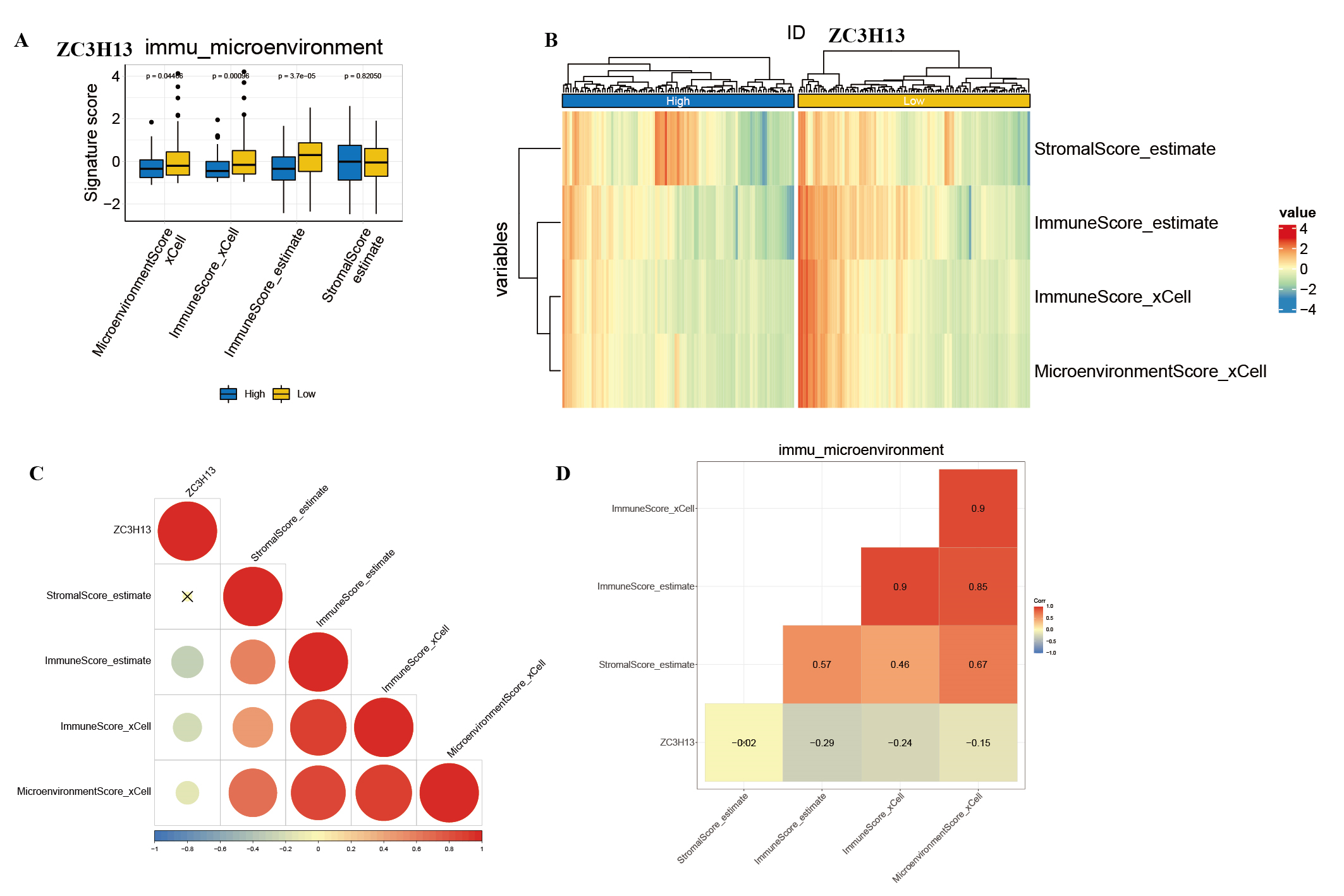

Supplement: Supplementary file 10 [file Image5.tif]
