# Supplementary material for: Angiogenesis-related gene signatures reveal the prognosis of cervical cancer based on single cell sequencing and co-expression network analysis
Source: Front Cell Dev Biol. 2023 Jan 12;10:1086835. doi: 10.3389/fcell.2022.1086835 (PMC9877352; doi:10.3389/fcell.2022.1086835)
Supplement: Supplementary file 2 [file Table1.docx]

| gene | Cell Type |
| --- | --- |
| CD28A | Lymphocytos |
| CD27 | Lymphocytos |
| PRF1 | Lymphocytos |
| CD163 | macrophages |
| FCGR2A | macrophages |
| COL1A2 | Fibroblasts |
| APOD | Fibroblasts |
| PECAM1 | Endothelial cells |
| EGFL7 | Endothelial cells |
| EMCN | Endothelial cells |
| ACTG2 | smooth muscle cells |
| EPCAM | Tumor/epithelial cells |
| CDH1 | Tumor/epithelial cells |
| CDKN2A | Tumor/epithelial cells |

**Table S1|Annotation of cell surface annotator genes**

| ID | Hazard_ratio | Low_CI | High_CI | p_value |
| --- | --- | --- | --- | --- |
| CELF2 | 0.926599 | 0.859165 | 0.999325 | 0.047989 |
| HLA.DPB1 | 0.996697 | 0.994186 | 0.999215 | 0.010159 |
| CD74 | 0.999746 | 0.999549 | 0.999944 | 0.011822 |
| TPM3 | 1.010388 | 1.003197 | 1.01763 | 0.004569 |
| AMD1 | 1.022631 | 1.005341 | 1.040218 | 0.010103 |
| ZMYM2 | 1.028183 | 1.006006 | 1.050849 | 0.012482 |
| ZC3H13 | 1.031757 | 1.008761 | 1.055277 | 0.006559 |
| TXNDC12 | 1.102507 | 1.0441 | 1.164182 | 0.000442 |

**Table S2| HR values of 8 genes in a prognostic model related to vascular neogenesis**

| ID | Coef |
| --- | --- |
| CD74 | -1.69E-05 |
| TPM3 | 0.002625 |
| ZC3H13 | 0.001495 |
| TXNDC12 | 0.044585 |
| CELF2 | -8.10E-04 |
| ZMYM2 | 0.003003 |
| HLA-DPB1 | -6.52E-04 |
| AMD1 | 0.001105 |

**Table S3| The coefficient of lasso regression of 8 model genes**

| \| ID \| Description \| pvalue \| p.adjust \| qvalue \| geneID \| one_type \| two_type \| \| --- \| --- \| --- \| --- \| --- \| --- \| --- \| --- \| \| hsa04612 \| Antigen processing and presentation \| 0.000941 \| 0.031986 \| 0.017825 \| HLA-DPB1/CD74 \| Organismal Systems \| Immune system \| \| hsa05152 \| Tuberculosis \| 0.004864 \| 0.082688 \| 0.04608 \| HLA-DPB1/CD74 \| Human Diseases \| Infectious disease: bacterial \| \| hsa05310 \| Asthma \| 0.019438 \| 0.089311 \| 0.049771 \| HLA-DPB1 \| Human Diseases \| Immune disease \| \| hsa05216 \| Thyroid cancer \| 0.023165 \| 0.089311 \| 0.049771 \| TPM3 \| Human Diseases \| Cancer: specific types \| \| hsa05330 \| Allograft rejection \| 0.023785 \| 0.089311 \| 0.049771 \| HLA-DPB1 \| Human Diseases \| Immune disease \| \| hsa05332 \| Graft-versus-host disease \| 0.025643 \| 0.089311 \| 0.049771 \| HLA-DPB1 \| Human Diseases \| Immune disease \| \| hsa04940 \| Type I diabetes mellitus \| 0.02688 \| 0.089311 \| 0.049771 \| HLA-DPB1 \| Human Diseases \| Endocrine and metabolic disease \| \| hsa00270 \| Cysteine and methionine metabolism \| 0.030584 \| 0.089311 \| 0.049771 \| AMD1 \| Metabolism \| Amino acid metabolism \| \| hsa04672 \| Intestinal immune network for IgA production \| 0.030584 \| 0.089311 \| 0.049771 \| HLA-DPB1 \| Organismal Systems \| Immune system \| \| hsa00330 \| Arginine and proline metabolism \| 0.031201 \| 0.089311 \| 0.049771 \| AMD1 \| Metabolism \| Amino acid metabolism \| \| hsa05320 \| Autoimmune thyroid disease \| 0.033048 \| 0.089311 \| 0.049771 \| HLA-DPB1 \| Human Diseases \| Immune disease \| \| hsa05168 \| Herpes simplex virus 1 infection \| 0.033886 \| 0.089311 \| 0.049771 \| HLA-DPB1/CD74 \| Human Diseases \| Infectious disease: viral \| \| hsa00480 \| Glutathione metabolism \| 0.034892 \| 0.089311 \| 0.049771 \| TXNDC12 \| Metabolism \| Metabolism of other amino acids \| \| hsa05416 \| Viral myocarditis \| 0.037346 \| 0.089311 \| 0.049771 \| HLA-DPB1 \| Human Diseases \| Cardiovascular disease \| \| hsa05321 \| Inflammatory bowel disease (IBD) \| 0.040407 \| 0.089311 \| 0.049771 \| HLA-DPB1 \| Human Diseases \| Immune disease \| \| hsa05140 \| Leishmaniasis \| 0.047114 \| 0.089311 \| 0.049771 \| HLA-DPB1 \| Human Diseases \| Infectious disease: parasitic \| \| KEGG \|  \|  \|  \|  \|  \|  \|  \| |
| --- | --- | --- | --- | --- | --- | --- | --- | --- | --- | --- | --- | --- | --- | --- | --- | --- | --- | --- | --- | --- | --- | --- | --- | --- | --- | --- | --- | --- | --- | --- | --- | --- | --- | --- | --- | --- | --- | --- | --- | --- | --- | --- | --- | --- | --- | --- | --- | --- | --- | --- | --- | --- | --- | --- | --- | --- | --- | --- | --- | --- | --- | --- | --- | --- | --- | --- | --- | --- | --- | --- | --- | --- | --- | --- | --- | --- | --- | --- | --- | --- | --- | --- | --- | --- | --- | --- | --- | --- | --- | --- | --- | --- | --- | --- | --- | --- | --- | --- | --- | --- | --- | --- | --- | --- | --- | --- | --- | --- | --- | --- | --- | --- | --- | --- | --- | --- | --- | --- | --- | --- | --- | --- | --- | --- | --- | --- | --- | --- | --- | --- | --- | --- | --- | --- | --- | --- | --- | --- | --- | --- | --- | --- | --- | --- |

**Table S4|** **Analysis of KEGG pathway of model genes**

| Term | P-value | Odds Ratio | Combined Score | Genes |
| --- | --- | --- | --- | --- |
| Penconazole CTD 00003093 | 0.005189 | 237.8571 | 1251.416 | TPM3 |
| bisacodyl MCF7 UP | 0.003171 | 30.51852 | 175.5917 | ZMYM2;AMD1 |
| Nickelous acetate CTD 00003684 | 0.024537 | 46.67681 | 173.0583 | TXNDC12 |
| Zinc sulfate CTD 00007264 | 0.005464 | 22.96737 | 119.6509 | HLA-DPB1;TXNDC12 |
| staurosporine MCF7 DOWN | 0.001686 | 17.96842 | 114.7381 | ZMYM2;AMD1;ZC3H13 |
| VALPROIC ACID CTD 00006977 | 0.010897 | 9.848543 | 44.50776 | CD74;ZMYM2;TPM3;CELF2;AMD1;HLA-DPB1;ZC3H13 |

**Table S5|** **Potential Small Molecule Drug Prediction**
