# Supplementary material for: Angiogenesis-related gene signatures reveal the prognosis of cervical cancer based on single cell sequencing and co-expression network analysis
Source: Front Cell Dev Biol. 2023 Jan 12;10:1086835. doi: 10.3389/fcell.2022.1086835 (PMC9877352; doi:10.3389/fcell.2022.1086835)
Supplement: Supplementary file 11 [file DataSheet1.docx]

**Supplementary Figure S1|Cell localization of marker-genes**

**Supplementary Figure S2|Comparison of immune cells and related marker genes in high-AGS group and low-AGS group. (A and B).** Differential expression of dendritic cells.  **(C and D).** Differential expression of marker genes of CD8 T effector cells.

**Supplementary Figure S3|Comparison of marker genes of DNA damage repair and immune checkpoint in high-AGS group and low-AGS group. (A and B).** Differential expression of marker genes of DNA damage repair. **(C and D).** Differential expression of marker genes of immune checkpoint.

**Supplementary Figure S4|Immune microenvironment scores of TXNDC12 in High-AGS Group and Low- AGS Group. (A and B).** Immune microenvironment scores, immune cells scores and stromal cells scores of TXNDC12. **(C and D)**. Correlation between TXNDC12 and three types of scores.

**Supplementary Figure S5|Immune microenvironment scores of ZC3H13 in High-AGS Group and Low- AGS Group. (A and B).** Immune microenvironment scores, immune cells scores and stromal cells scores of ZC3H13. **(C and D)** Correlation between ZC3H13 and three types of scores.

**Supplementary Figure S6| Data quality control and evaluation. (A and B).** Quality control for the single-cell sequencing data. **(C)**. The selection of soft threshold. **(D).** The division of modules with merged similarity. **(E)**. The two-dimensional diagram of PCA analysis in TCGA-CESC cohort. **(F)**. The two-dimensional diagram of PCA analysis in GSE44001. **(G)** Volcano plot of differential proteins between subgroups. **(H and I).** Evaluation the accuracy by ridge regression and lasso regression in the IOBR algorithm. **(J)**. Evaluation survival status by ridge regression in the IOBR algorithm.

**Supplementary Figure S7|Heatmap of immune cell infiltration in high-AGS group and low- AGS group of TCGA-CESC cohort**

**Supplementary Figure S8|Expression of differential mutation genes between high and low AGS groups**

**Supplementary Figure S9|** **Survival curves and ROC curves for TCGA internal train set, test set, and GEO external validation set. (A, B, C, G).** Heat map, survival curve, ROC curve, PCA analysis of TCGA internal train set. **(D, E, F, H).** Heat map, survival curve and ROC curve, PCA analysis of TCGA internal test set. **(I, J).** Survival curves and ROC curves for GEO external validation set. Grouping based on cut-off values.
